# RNA-sequencing based first choice of treatment and determination of risk in multiple myeloma

**DOI:** 10.3389/fimmu.2023.1286700

**Published:** 2023-11-15

**Authors:** Martina Emde-Rajaratnam, Susanne Beck, Vladimir Benes, Hans Salwender, Uta Bertsch, Christoph Scheid, Mathias Hänel, Katja Weisel, Thomas Hielscher, Marc S. Raab, Hartmut Goldschmidt, Anna Jauch, Ken Maes, Elke De Bruyne, Eline Menu, Kim De Veirman, Jérôme Moreaux, Karin Vanderkerken, Anja Seckinger, Dirk Hose

**Affiliations:** ^1^Department of Hematology and Immunology, Myeloma Center Brussels & Labor für Myelomforschung, Vrije Universiteit Brussel (VUB), Jette, Belgium; ^2^Universitätsklinikum Heidelberg, Molekularpathologisches Zentrum, Heidelberg, Germany; ^3^Europäisches Laboratorium für Molekularbiologie, GeneCore, Heidelberg, Germany; ^4^Asklepios Tumorzentrum Hamburg, AK Altona and St. Georg, Hamburg, Germany; ^5^Universitätsklinikum Heidelberg, Medizinische Klinik V, Heidelberg, Germany; ^6^Department I of Internal Medicine, University of Cologne, Cologne, Germany; ^7^Department of Internal Medicine III, Klinikum Chemnitz GmbH, Chemnitz, Germany; ^8^Department of Oncology, Hematology and Bone Marrow Transplantation with Section of Pneumology, University Medical Center Hamburg-Eppendorf, Hamburg, Germany; ^9^Deutsches Krebsforschungszentrum, Abteilung für Biostatistik, Heidelberg, Germany; ^10^Nationales Centrum für Tumorerkrankungen, Heidelberg, Germany; ^11^Universität Heidelberg, Institut für Humangenetik, Heidelberg, Germany; ^12^Institute of Human Genetics, UMR 9002 CNRS-UM, Montpellier, France

**Keywords:** multiple myeloma, immunotherapeutic targets, personalized treatment, risk-adapted treatment, RNA-sequencing, survival, proliferation

## Abstract

**Background:**

Immunotherapeutic targets in multiple myeloma (MM) have variable expression height and are partly expressed in subfractions of patients only. With increasing numbers of available compounds, strategies for appropriate choice of targets (combinations) are warranted. Simultaneously, risk assessment is advisable as patient’s life expectancy varies between months and decades.

**Methods:**

We first assess feasibility of RNA-sequencing in a multicenter trial (GMMG-MM5, n=604 patients). Next, we use a clinical routine cohort of untreated symptomatic myeloma patients undergoing autologous stem cell transplantation (n=535, median follow-up (FU) 64 months) to perform RNA-sequencing, gene expression profiling (GEP), and iFISH by ten-probe panel on CD138-purified malignant plasma cells. We subsequently compare target expression to plasma cell precursors, MGUS (n=59), asymptomatic (n=142) and relapsed (n=69) myeloma patients, myeloma cell lines (n=26), and between longitudinal samples (MM vs. relapsed MM). Data are validated using the independent MMRF CoMMpass-cohort (n=767, FU 31 months).

**Results:**

RNA-sequencing is feasible in 90.8% of patients (GMMG-MM5). Actionable immune-oncological targets (n=19) can be divided in those expressed in all normal and >99% of MM-patients (CD38, SLAMF7, BCMA, GPRC5D, FCRH5, TACI, CD74, CD44, CD37, CD79B), those with expression loss in subfractions of MM-patients (BAFF-R [81.3%], CD19 [57.9%], CD20 [82.8%], CD22 [28.4%]), aberrantly expressed in MM (NY-ESO1/2 [12%], MUC1 [12.7%], CD30 [4.9%], mutated BRAF V600E/K [2.1%]), and resistance-conveying target-mutations e.g., against part but not all BCMA-directed treatments. Risk is assessable regarding proliferation, translated GEP- (UAMS70-, SKY92-, RS-score) and *de novo* (LfM-HRS) defined risk scores. LfM-HRS delineates three groups of 40%, 38%, and 22% of patients with 5-year and 12-year survival rates of 84% (49%), 67% (18%), and 32% (0%). R-ISS and RNA-sequencing identify partially overlapping patient populations, with R-ISS missing, e.g., 30% (22/72) of highly proliferative myeloma.

**Conclusion:**

RNA-sequencing based assessment of risk and targets for first choice treatment is possible in clinical routine.

## 1 Introduction

Multiple myeloma (MM) is a malignant hematological disease characterized by accumulation of clonal plasma cells in the bone marrow. Clinical signs and symptoms relate to displacement of normal hematopoiesis, generation of osteolytic bone disease, and renal impairment (1). Treatment is initiated if such end organ damage is present, or its occurrence imminent as predicted by biomarkers (2). Treatment has significantly improved during the preceding four decades due to introduction of small molecules and immune-oncological drugs into routine clinical practice, including monoclonal antibodies targeting CD38 (daratumumab, isatuximab) (3, 4), SLAMF7/CS1 (elotuzumab) (5), GPRC5D (talquetamab) (6), and different BCMA-targeting strategies, i.e. the anti-BCMA antibody-drug conjugate (ADC) belantamab mafodotin (7), the BCMA CAR-T cell products idecabtagen vicleucel (8) and ciltacabtagene autoleucel (9), and the BCMA T-cell bispecific antibodies teclistamab and elrantamab (10, 11).

MM is treated by combination treatment whenever possible (12, 13): effective quadruple combinations followed by autologous stem cell transplantation (ASCT) (e.g., GRIFFIN-trial) increases response rates from about 1/3 for single agents (14–20) to almost 100% of patients (13, 21). At the same time, it is not possible to predict response to small molecules at a clinically applicable level (22, 23), despite we and others have published factors associated with response such as an eight-gene-signature for thalidomide and dexamethasone induction followed by ASCT (24), MCT1 for lenalidomide (25), or BCL2 or BCL2/BCL2L1-ratio for venetoclax treatment (26–28). Exemplary exception is the presence of the BRAF V600E/K mutation, shown to be successfully targetable e.g. by vemurafenib (29).

The situation is different for immune-oncological therapies in development or recently approved. Bispecific antibodies or CAR-T cells against e.g., BCMA, GPRC5D, or FCR5H show single agent remission rates of 60-80% (8–10, 30–38). Most importantly, they have a distinct molecularly assessable target. Targets like CD38, BCMA or GPRC5D have variable expression height (39–41), and several are expressed in subfractions of patients only, e.g. CD19 or NY-ESO1/2 (42).

Presence or absence and height of target expression is an evident selection criterion for treatment choice, as shown, e.g., for CD38 (43) and GPRC5D (41), or a potential use of γ-secretase inhibitors (e.g. crenigacestat) (44) in case of low BCMA-expression. Recent studies identified BCMA-mutations conveying resistance to only part of respective T-cell bispecific antibodies and CAR-T treatments, suggesting the possibility to switch within BCMA-targeting agents to a different compound (45).

As individual myeloma patients have life expectancies varying from months to decades, it is helpful for patient counselling and risk-adapted treatment strategies to assess risk. Current gold standard is the revised-ISS staging system (R-ISS) incorporating clinical factors (serum beta-2-microglobulin and albumin) and molecular alterations in malignant plasma cells (deletion 17p13, t(4;14), t(14;16) assessed by interphase fluorescence *in situ* hybridization [iFISH]) (46). Innovative methods include transcriptome profiling by DNA-microarrays (GEP) or RNA-sequencing, assessing plasma cell proliferation (47) or “scoring” over genes associated with prognosis (48–52).

In this manuscript, we first assess applicability of RNA-sequencing in the GMMG-MM5 multicenter phase III clinical trial setting (604 patients) based on our low-input RNA-sequencing protocol (53). Secondly, we use a clinical routine cohort of 535 patients investigated by RNA-sequencing, GEP, and multi-parameter iFISH. We assess presence and expression height of actionable immunological targets, mutated BRAF V600E/K, and potential resistance-conveying mutations of these genes. We aim at delineating in what percentage of patients an “educated first choice” is possible on the simulated background of all immune-oncological compounds approved or in clinical trials (clinicaltrials.gov “active” or “completed”, **Table 1**) being available. We further compare expression of identified targets in MM to plasma cell precursor populations, early-stage plasma cell dyscrasias, as well as to relapsed patients. The latter to first delineate whether a target might be specifically suited for early (e.g., CD19, lost in later myeloma stage) or late (e.g., cancer testis antigens, gained) treatment. Third, we take a fresh look at risk assessment by transferring proliferation and microarray-based risk scores to RNA-sequencing and establish a *de novo* risk score by RNA-sequencing (termed LfM-HRS). Findings are validated in the independent MMRF CoMMpass-cohort (n=767 patients).

## 2 Methods

### 2.1 Feasibility of RNA-sequencing based on the GMMG-MM5 trial

Patients (n=604) were included in the prospective GMMG MM5-trial (51, 54) between July 2010 and November 2013 in 31 transplant centers and 75 associated sites throughout Germany. As per protocol, bone marrow aspirates at study inclusion, i.e., before treatment, were available for n=573 patients (94.9%), of whom we were able to successfully perform plasma cell purification followed by quality control using flow cytometry for n=559 patients (97.6%). The 31 lacking samples (5.1%) were due to patients declining the bone marrow aspiration (2.5%) or punctio sicca (2.5%). Median purity according to CD38/CD138 double staining was 87.9% with a median cell number of 1.2 × 10^6^ cells (51).

iFISH using cytospins from CD138-purified plasma cells was performed centrally (Multiple Myeloma Research Laboratory and Department of Human Genetics, Heidelberg). Data could be obtained for 556/573 patients with available bone marrow aspirates (97%) and 556/559 patients with available CD138-purified plasma cells, respectively (99.5%). The median proportion of malignant plasma cells determined per iFISH, i.e., the highest percentage of a chromosomal aberration, was 95% (51).

Samples for RNA-extraction followed by quality control were collected over two weeks and subjected to GEP by DNA-microarrays. In total, n=458 transcriptome datasets are available, i.e., 81.9% of patients with available CD138-purified plasma cells. Of these, two patients were excluded from further analysis for not fulfilling the trial’s inclusion criteria. Gene expression profiling could not be performed in 53 cases due to low RNA quality (9.5%) and further 48 cases (8.6%) in which not enough RNA was available (51).

Using our standardized RNA-sequencing protocol (53) (see below), RNA-sequencing was possible in all patients considered as with “too low” amount of RNA for GEP. In total, in 506/559 patients with available CD138-purified plasma cells (90.5%) and 506/604 patients of the intention to treat population (83.8%).

### 2.2 Feasibility of RNA-sequencing in clinical routine

Consecutive patients with monoclonal gammopathy of unknown significance (MGUS; n=52), asymptomatic (AMM; n=142), symptomatic, therapy-requiring myeloma (MM; n=535), relapsed myeloma (MMR; n=69), as well as healthy donors (n=10) as comparators were included in the study approved by the ethics committee (#229/2003, #S-152/2010) after written informed consent. For patient characteristics, see **Supplementary Table S1**.

Normal bone marrow plasma cells (BMPCs) and myeloma cells were purified using anti-CD138 microbeads (Miltenyi Biotec, Bergisch Gladbach, Germany). Peripheral blood CD27^+^ memory B-cells (n=4) were FACS-sorted and polyclonal plasmablasts (n=4) were *in vitro* differentiated as described (55). A total of 26 human myeloma cell lines (HMCL) were included (see **Supplementary Appendix**).

RNA-sequencing data of 52/142/535/69 patients with MGUS/AMM/MM/MMR and 26 HMCLs were used. GEP-data of 534 patients with MM were used for score definition and validation (i.e., translation of LfM-HRS into a GEP-based score).

### 2.3 Independent validation of risk assessment and target identification

Independent validation of risk assessment and target identification was performed using the Multiple Myeloma Research Foundation (MMRF) CoMMpass trial (NCT01454297), i.e., n=767 previously untreated myeloma patients with RNA-sequencing data available (release 13).

### 2.4 Analysis of gene expression

#### 2.4.1 RNA-extraction

RNA was extracted using the Qiagen AllPrep DNA/RNA kit (Qiagen, Hilden, Germany) according to the manufacturer’s instructions. Quality control and quantification was performed using an Agilent 2100 bioanalyzer (Agilent, Frankfurt, Germany).

#### 2.4.2 RNA-sequencing

RNA-sequencing was performed as published (53). In brief, full-length double-stranded cDNA was generated from 5 ng of total RNA and amplified using the SMARTer Ultra Low RNA Kit (Illumina, San Diego, CA, USA). Library preparation was performed from 10 ng of fragmented cDNA using the NEBNext Chip-Seq Library Prep protocol (New England BioLabs, Ipswich, MA, USA). Libraries were sequenced on an Illumina Hiseq2000 with 2x50-bp paired-end reads. RNA-sequencing expression data are deposited in the European nucleotide archive (PRJEB37100, PRJEB36223).

#### 2.4.3 Gene expression profiling

Gene expression profiling (GEP) using U133 2.0 plus arrays (Affymetrix, Santa Clara, CA, USA) was performed as published (39, 47, 56–59). Expression data are deposited in ArrayExpress (E-MTAB-4715, E-MTAB-4717, E-MTAB-5212, E-TABM-937, and E-TABM-1088).

### 2.5 iFISH

iFISH analysis was conducted on CD138-purified plasma cells using probes for chromosomes 1q21, 5p15, 5q31 or 5q35, 8p21, 9q34, 11q22.3 or 11q23, 13q14.3, 15q22, 17p13, 19q13, IgH-breakapart, as well as translocations t(4;14)(p16.3;q32.3), t(11;14)(q13;q32.3), and t(14;16)(q32.3;q32) according to the manufacturer’s instructions (Kreatech, Amsterdam, The Netherlands and MetaSystems, Altlussheim, Germany) and data were analyzed as published (60).

### 2.6 Statistical analysis

GEP and RNA-seq analysis were performed as previously described (42, 51, 58) (for details and modifications, see **Supplementary Appendix**). Computations were performed using R (https://www.r-project.org/) and Bioconductor (https://www.bioconductor.org/) versions 3.3.2 and 3.4 (score implementation) and versions 3.4.4 and 3.6 (further analysis).

Overall (OS) and event-free (EFS) survival were investigated for symptomatic multiple myeloma undergoing high-dose therapy using Cox’s proportional hazard model as published (61).

Survival curves were computed with nonparametric survival estimates for censored data using the Kaplan-Meier method (62). Difference between the curves were tested using the G-rho Log-rank test (63). Wilcoxon ranks sum test and Jonckheere-Terpstra test were used to investigate differences in gene expression between groups and to test for an ordered alternative hypothesis within independent samples (between participants) design, respectively. A Chi-squared test for trend in proportion (Cochran Armitage trend test) was used for comparison of presence of expression from MGUS to AMM to MM to MMR and AMM to MM to MMR, respectively. For comparison of expression in longitudinal samples (MM vs. MMR), a paired Wilcox-test was performed.

**Figure 1:**
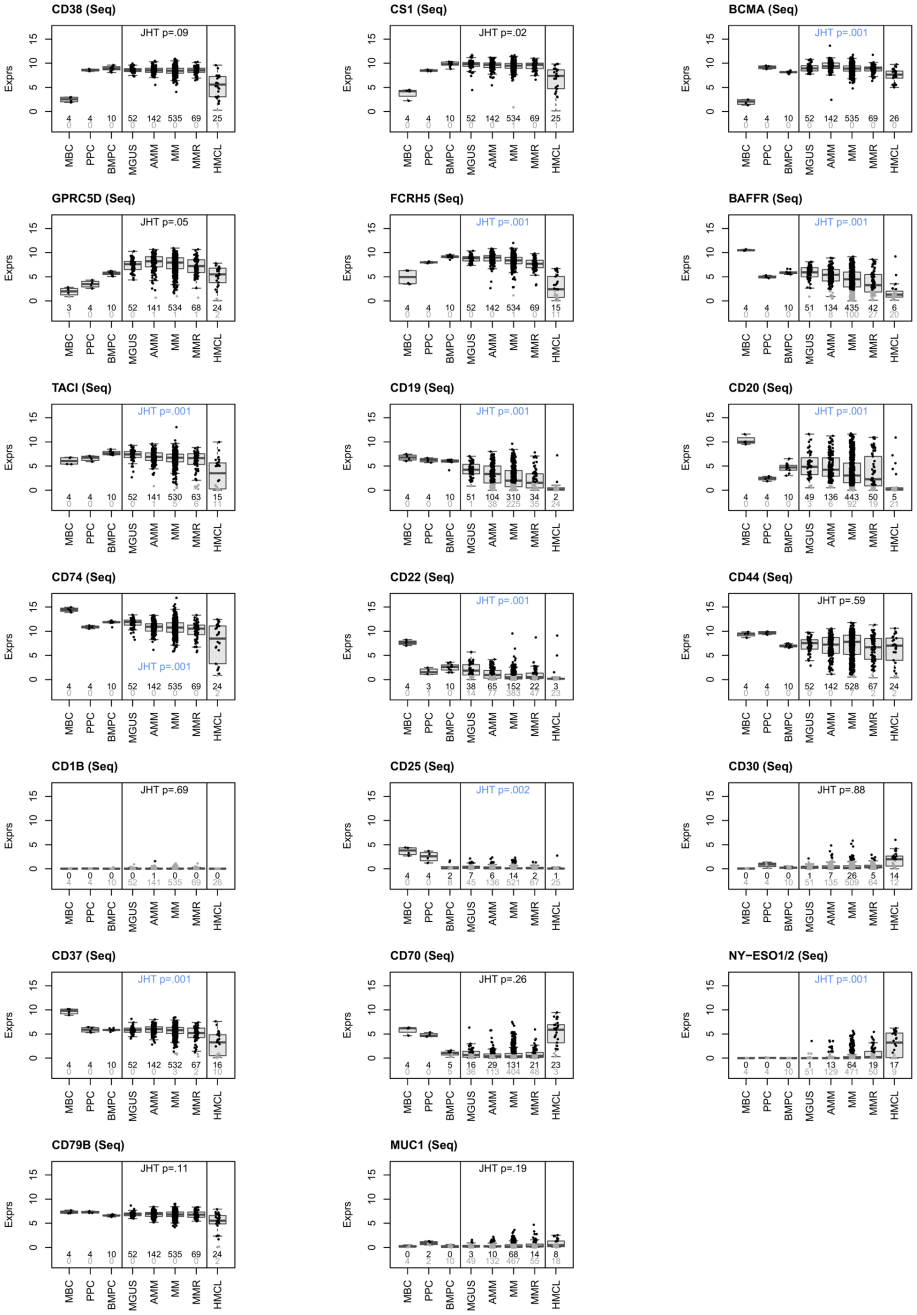
RNA-sequencing based assessment of immune-oncological actionable targets depicted in **Figure 4**. Expression height in malignant plasma cells from MGUS-, asymptomatic (AMM), symptomatic (MM) and relapsed myeloma patients (MMR) in comparison to normal bone marrow plasma cells (BMPC), memory B-cells (MBC), proliferating plasmablasts (PPC) and human myeloma cell lines (HMCL). Targets can be divided in those expressed in all normal and (almost all, >99%) malignant plasma cells (n=10; CD38, SLAMF7 (CS1), BCMA, GPRC5D, FCRH5, TACI, CD74, CD44, CD37 and CD79B), those constitutively expressed in all normal plasma cells with expression lost in a subfraction of malignant plasma cells (n=4; BAFF-R [81.3%], CD19 [57.9%], CD20 [82.8%], CD22 [28.4%]), and targets aberrantly expressed in malignant plasma cells, i.e., not expressed in BMPC, (n=3; NY-ESO1/2 [12%], MUC1 [12.7%], CD30 [4.9%]). Some suggested targets are not expressed (CD1B) or at a very low level in normal and malignant plasma cells (CD25 [2.6%]). Black and grey color of data points and corresponding numbers indicate “presence” and “absence” of expression, respectively. See **Table 2** for numerical depiction and details. “Present” expression by RNA-sequencing is defined as presence of at least one read count per million (CPM) per 1000 bp. Gene length is defined as median transcript length. Significant difference for higher (all other genes)- or lower expression (MUC1, NY-ESO1) of genes from MGUS to AMM to MM to MMR is assessed by Jonckheere-Terpstra Test (JHT). Exploratory *P*-values given. P-Values remaining significant after Benjamini-Hochberg adjustment for multiple testing are depicted in blue color. Note: part of expression data for BCMA and CD38 have previously been published (39, 58). For a comparison from AMM to MM to MMR, see **Supplementary Figure S2**.

**Figure 2:**
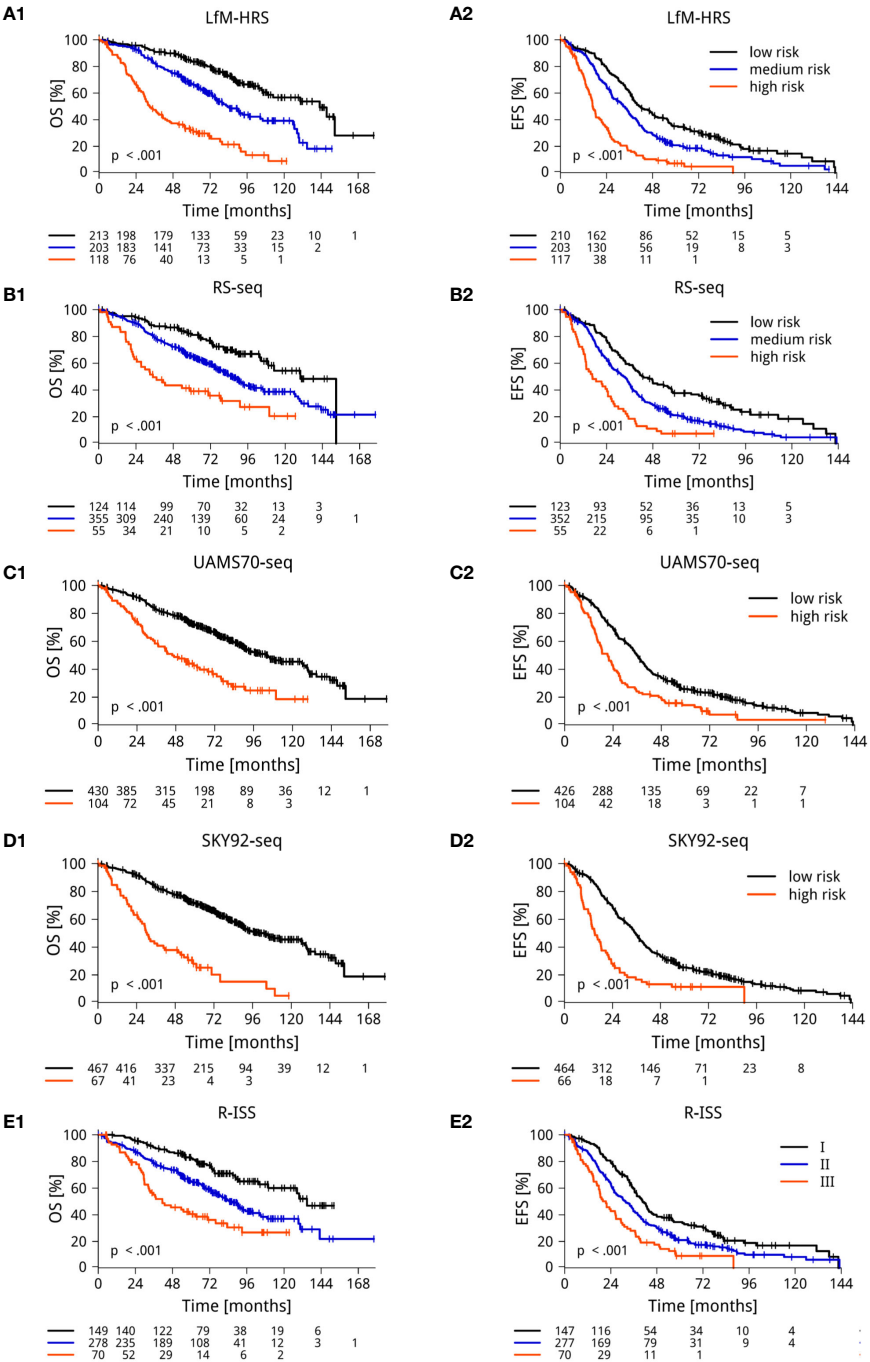
RNA-sequencing based determination of risk. **(A)** *De novo* generated RNA-sequencing-based scores for risk (LfM-HRS) delineates 3 groups with significantly different overall (OS) (**A1**) and event-free (EFS) (**A2**) survival. **(B-D) “GEP”-scores** translated into RNA-sequencing. The scores of the Universities of Heidelberg and Montpellier (RS-score) **(B)**, the University of Arkansas Medical School (UAMS70) **(C)**, and the Erasmus Medical Center (SKY92), **(D)** in each case delineate symptomatic myeloma patients with significantly different EFS and OS. **(E)**. The current clinical gold standard (revised ISS-score) delineates three groups of 30%, 56% and 14% of 535 patients with significantly different OS (**E1**) and EFS (**E2**). Depicted are Kaplan Maier curves with log-rank based P-value and patients at risk. *P*-values were adjusted for multiple testing using Benjamini-Hochberg correction. For validation of RNA-sequencing based scores on the independent CoMMpass-cohort, see **Supplementary Figure S5**.

**Figure 3:**
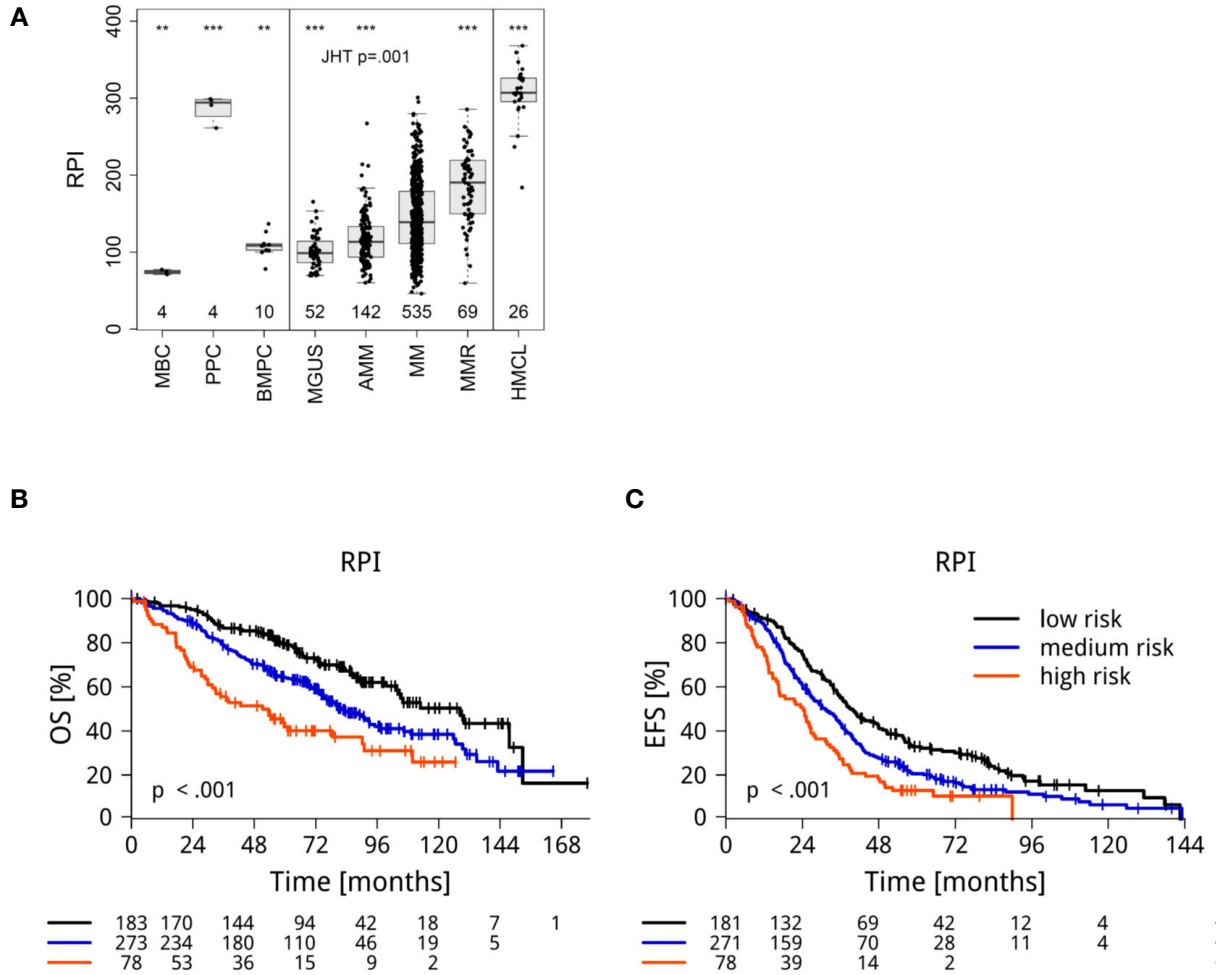
RNA-sequencing based determination of proliferation (RPI) **(A)** of malignant plasma cells from MGUS-, asymptomatic (AMM), symptomatic (MM) and relapsed myeloma patients (MMR) in comparison to normal bone marrow plasma cells (BMPC), memory B-cells (MBC), proliferating plasmablasts (PPC), and human myeloma cell lines (HMCL). Significant differences in comparison to MM are depicted by asterisks (*** P<.001, ** P<0.01). **(B)** Overall survival and **(C)** event- free survival. Depicted are Kaplan Maier curves with log-rank based *P*-value and patients at risk.

Effects were considered statistically significant if the P-value of corresponding statistical tests was below 5%. For comparisons of parameters (**Figure 1**) and regarding OS and EFS (**Figures 2**, **3**; **Supplementary Figures S3, S4, S5**), adjustment for multiple testing was made using the Benjamini-Hochberg correction. Adjustment was applied separately for the two cohorts assessed.

## 3 Results

### 3.1 RNA-sequencing assisted educated first choice of immune-oncological actionable targets and BRAF V600E/K mutation

**Figure 4:**
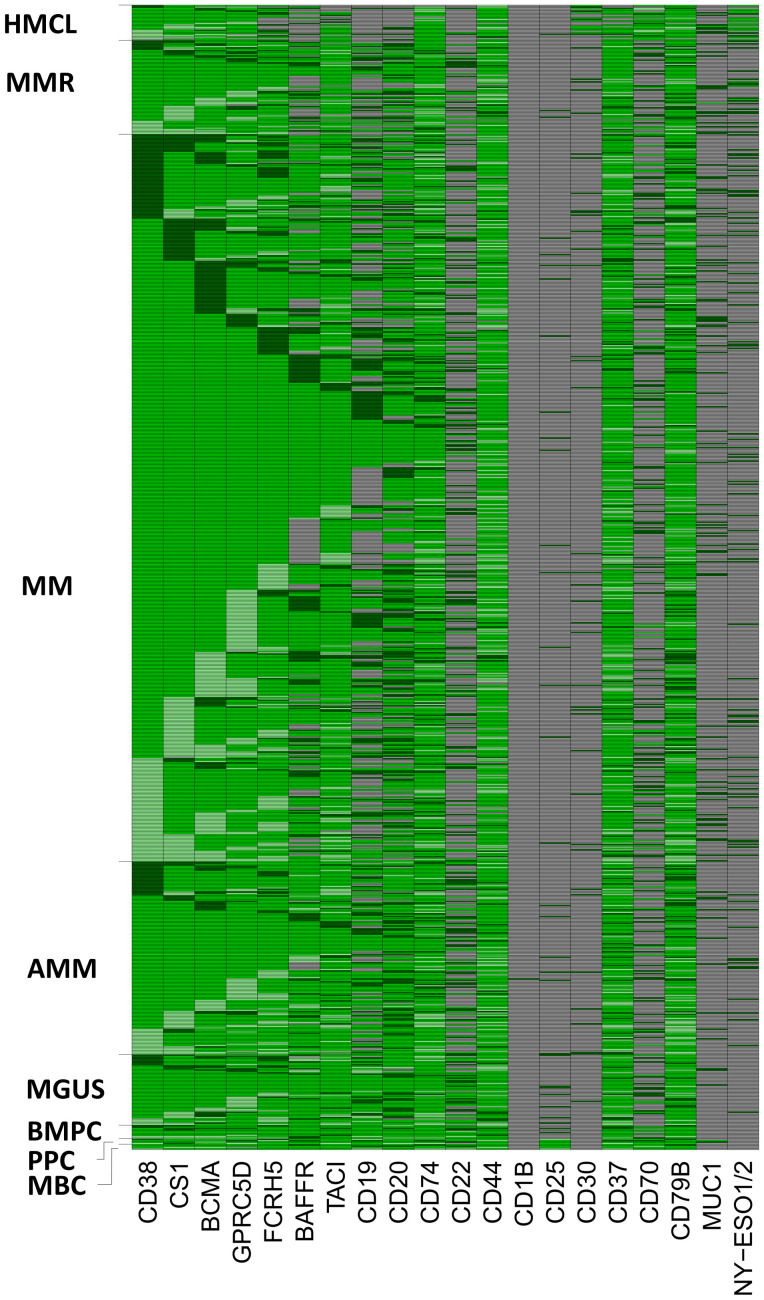
RNA-sequencing assisted educated first choice of 20 immune-oncological actionable targets (IOnc-advisor). Targets can be divided in those expressed in all normal bone marrow plasma cells [BMPC] and (almost all, >99%) malignant plasma cells (MMC) from MGUS-, asymptomatic (AMM), symptomatic (MM) and relapsed myeloma patients (MMR) [n=10; CD38, SLAMF7 [CS1], BCMA, GPRC5D, FCRH5, TACI, CD74, CD44, CD37 and CD79B], those constitutively expressed in all normal plasma cells with expression lost in a subfraction of malignant plasma cells (n=4; BAFF-R [81.3%], CD19 [57.9%], CD20 [82.8%], CD22 [28.4%]), and targets aberrantly expressed in malignant plasma cells, i.e., not expressed in BMPCs (n=3; NY-ESO1/2 [12%], MUC1 [12.7%], CD30 [4.9%]). Some suggested targets are not expressed (CD1B) or at a very low level in normal and malignant plasma cells (CD25 [2.9%]). Memory B-cells (MBC), proliferating plasmablasts (PPC) and human myeloma cell lines (HMCL) were used as comparators. Grey color indicates absence of target expression. Green color indicates expression. Target overexpression in comparison to the median expression within the respective population (BMPC or MMC) ± one standard-deviation is depicted in light (lower expression) and dark (higher expression) green. See **Table 2** for numerical depiction and details.

We first created a list of potentially actionable immune-oncological targets with either drugs approved in MM (CD38, SLAMF7 [CS1], BCMA), or ADC, antibody-radio-conjugates, bispecific antibodies, or CART-products in trials (clinicaltrial.gov (accessed January 14^th^, 2022, updated July 24^th^, 2023, **Table 1**) for i) patients with multiple myeloma, identifying GPRC5D, FCRH5, BAFF-R, TACI, CD19, CD20, CD22, CD74, CD44v6 and CD1b, ii) B-cell lymphoma, i.e. CD25, CD30, CD37, CD70, CD79b, and iii) solid oncology regarding antigens for which expression in a subfraction of myeloma patients has been reported [NY-ESO1 (42), MUC1 (64)], (**Figure 4**, **Table 1**, **2**). As example for targetable mutations, we included the BRAF V600E/K mutation. We termed this assessment "immune oncology advisor" (IOnc-advisor) in analogy to other decision tools.

**Table 1:** List of potentially actionable targets.

TARGET	DRUG				TRIAL	
	class	type	INN-name/name	manufacturer	trial	status
**CD38**	**TCB**	CD38xCD3 bispecific antibody	ISB 1342	Ichnos	NCT03309111	active
		CD38xCD3 bispecific antibody	Y150	Wuhan YZY Biopharma Co. Ltd.	NCT05011097	active
	**ADC**	CD38-Duostatin 5.2 ADC	STI-6129	Sorrento Therapeutics	NCT05308225	active (not recruiting)
	**DART**	allogeneid CD38 DART	STI-1492 DAR-T cells	Sorrento Therapeutics	NCT05007418	active
**SLAMF7 (CS1)**	**CART**	SLAMF7xCD3 allogeneic CART	UCARTCS1A/SLAMF7	Cellectis S.A.	NCT04142619	active
		SLAMF7xCD3 CART	CS1-CAR T/SLAMF7 autologous	City of Hope Medical Center & NCI, USA	NCT03710421	active
**BCMA**	**ADC**	BCMA-MMAF ADC	Belantamab mafodotin (GSK2857916)	GlaxoSmithKline	approved (MMR)	approved (MMR)
	**TCB**	BCMAxCD3 bispecific antibody	Alnuctamab (CC-93269)	BMS	NCT03486067	active
		BCMAxCD3 bispecific antibody	TNB-383B (ABBV-383)	AbbVie, TeneoOne	NCT03933735	active (not recruiting)
		BCMAxCD3 bispecific antibody	Elranatamab (PF-06863135)	Pfizer	e.g., NCT05090566	active
		BCMAxCD3 bispecific antibody	Linvoseltamab (REGN5458)	Regeneron Pharmaceuticals	e.g., NCT05137054	active
		BCMAxCD3 bispecific antibody	Teclistamab (JNJ-64007957)	JNJ	approved (MMR)	approved (MMR)
	**CART**	BCMA-CART	Idecabtagene vicleucel	BMS	approved (MMR)	approved (MMR)
		BCMA-CART	Ciltacabtagene autoleucel (JNJ-68284528)	JNJ	approved (MMR)	approved (MMR)
**GPCR5D**	**TCB**	GPCR5DxCD3 bispecific antibody	Talquetamab (JNJ-7564)	JNJ	e.g., NCT05552222	active
		GPCR5DxCD3 bispecific antibody	Forimtamig (RG2634/RO7425781)	Roche	NCT04557150	active
		GPCR5DxCD3 bispecific antibody	QLS32015	Qilu Pharmaceutical	NCT05920876	active
	**CART**	GPCR5D-CART	MCARH109	Memorial Sloan Kettering Cancer Center, USA	NCT05431608	active
		GPCRSD-CART	GPCR5D CAR-T cells	XuYan, Institute of Hematology & Blood Diseases Hospital, China	NCT05749133	active
**FcRH5**	**TCB**	FcRH5xCD3 bispecific antibody	Cevostamab (BFCR4350A)	Roche	e.g., NCT03275103	active
**BAFFR**	**CART**	BAFFR-CART	BAFFR CAR-T cells	PeproMene Bio, Inc. & City of Hope, USA	NCT04690595	active
**TACI & BCMA**	**CART**	APRIL-CART	APRIL CAR-T cells	Yake Biotechnology Ltd	NCT04657861	active
**CD19**	**TCB**	CD19xCD3 BITE	Blinatumumab	Amgen	approved (not MM)	approved (not MM)
	**CART**	CD19/BCMA dual CART	CART-BCMA huCART19/BCMA & CD19	Penn State University, USA	NCT03549442	active (not recruiting)
		CD19/BCMA dual CART	GC012F	Shanghai Changzheng Hospital, China	NCT04935580	active
**CD20**	**TCB**	CD20xCD3 bispecific antibody	Odronextamab (REGN1979)	Regeneron Pharmaceuticals	e.g., NCT02651662	active
**CD74**	**ADC**	CD74ADC	BN301 (STRO-001)	Sutro Biopharma, Inc./BioNova Pharmaceuticals	e.g., NCT05611853	active
**CD22**	**ADC**	CD22-ADC	Inotuzumab ozogamicin	Pfizer	approved (not MM)	approved (not MM)
	**CART**	CD22-CART	CD22 CAR-T cells	Stanford University, USA	e.g., NCT04088890	active
**CD44v6**	**CART**	CD44v6-CART	4SCAR-CD44v6 CAR-T cells	Shenzhen Geno-Immune Medical Institute, China	NCT04427449	active
**CD1b**	**TCB**	CD1bxV62-gammabody	LAVA-051 (gammabody, bispecific VHH)	Lava Therapeutics	NCT04887259	active (not recruiting)
**CD25**	**ARC**	90Y-CD25 Mab	90Y-DOTA-anti-CD25 basiliximab	City of Hope & NCI, USA	e.g., NCT05139004	active
**CD30**	**ADC**	CD30-MMAE ADC	Brentuximab vedotin	Seagen/Takeda Pharmaceutical Company	approved (not MM)	approved (not MM)
	**CART**	CD30-CART	CD30 CAR-T cells	Tessa Therapeutics	NCT04526834	active (not recruiting)
**CD37**	**CART**	CD37-CART	CAR-37 T-cells	Massachusetts General Hospital, USA	NCT04136275	active
**CD70**	**CART**	CD70-CART	CD70 CAR T-cells	Zhejiang University, China	NCT04662294	active
**CD79b**	**TCE**	CD79bxCD20xCD3 trispecific antibody	JNJ-80948543	JNJ	NCT05424822	active
	**CART**	CD79b-CART	JV-213	M.D. Anderson Cancer Center, USA	NCT05773040	active
**NY-ESO1**	**CART**	NY-ESO1-CART	TCRT-ESO-A2	Athenex, Inc.	NCT03462316	active (not recruiting)
**MUC1**	**CART**	MUC1-CART	MUC1-CART	Zhejiang University, China	NCT03633773	active

Review of clincaltrials.gov (January 14, 2022, updated July 24, 2023). Depicted are 20 targets with available antibody-drug conjugates (ADC) or antibody-radionuclide-conjugates (ARC), CART, or T-cell bispecific antibodies (TCB) with active trials in multiple myeloma, B-cell malignancies, or solid oncology (in case of MUC1, NY-ESO1, for which expression in a subfraction of myeloma patients has been reported). For each target, exemplary trials are stated (for ease of depiction, non-comprehensive list).

**Table 2:** RNA-sequencing based assessment of 20 suggested immune-oncological actionable targets (IOnc-advisor).

TARGET	Expression BMPC						Expression MMC					
	n	%	median	min	max	SDV	n	%	median	min	max	SDV
**CD38**	10	100	9	8,1	9,6	0,48	535	100	8,5	4,1	10,9	0,83
**CS1**	10	100	9,8	8,8	10,3	0,47	534	99,8	9,5	0,9	11,7	0,85
**BCMA**	10	100	8,2	7,8	8,6	0,22	535	100	8,9	4,8	11,7	0,9
**GPRC5D**	10	100	5,8	5,1	6,2	0,37	534	99,8	8	1	10,9	1,74
**FCRH5**	10	100	9,1	8,5	9,6	0,29	534	99,8	8,4	1,1	12	1,12
**BAFFR**	10	100	5,8	5,5	6,6	0,39	435	81,3	4,5	0	9,1	2,12
**TACI**	10	100	7,6	7,3	8,5	0,41	530	99,1	6,7	0	13,1	1,49
**CD19**	10	100	6,1	4,1	6,3	0,66	310	57,9	2	0	9,6	2,2
**CD20**	10	100	4,7	3	6,5	1,02	443	82,8	3	0	11,8	3,02
**CD74**	10	100	11,9	10,8	12,2	0,38	535	100	10,8	5,8	16,9	1,52
**CD22**	10	100	2,6	1,5	3,6	0,73	152	28,4	0,5	0	9,5	0,92
**CD44**	10	100	7	6,6	7,5	0,29	528	98,7	7,8	0,5	11,8	2,59
**CD1B**	0	0	0	0	0,4	0,11	0	0	0	0	1,1	0,14
**CD25**	2	20	0,1	0	1,7	0,62	14	2,6	0,1	0	2,3	0,33
**CD30**	0	0	0,2	0	0,6	0,19	26	4,9	0,2	0	5,8	0,67
**CD37**	10	100	5,8	5,4	6,3	0,23	532	99,4	5,8	0,7	8,4	1,1
**CD70**	5	50	1,1	0,2	1,6	0,47	131	24,5	0,3	0	7,5	1,2
**CD79B**	10	100	6,6	6,3	6,9	0,15	535	100	6,8	4,2	9	0,72
**MUC1**	0	0	0,2	0	0,6	0,22	68	12,7	0,2	0	3,6	0,54
**NY-ESO1/2**	0	0	0	0	0	0	64	12	0	0	5,6	0,92

Targets can be divided in those expressed in all normal bone marrow plasma cells (BMPC) and (almost all, >99%) malignant plasma cells from therapy-requiring multiple myeloma patients (MMC; n=10, i.e., CD38, SLAMF7 [CS1], BCMA, GPRC5D, FCRH5, TACI, CD74, CD44, CD37 and CD79B), those constitutively expressed in all normal plasma cells with expression lost in a subfraction of malignant plasma cells (n=4, i.e., BAFF-R [81.3%, CD19 [57.9%], CD20 [82.8%], CD22 [28.4%]), and targets aberrantly expressed in malignant plasma cells (i.e., not expressed in BMPC) (n=3, i.e., NY-ESO1/2 [12%], MUC1 [12.7%], CD30 [4.9%]). CD70 is expressed in a subfraction of BMPC (50%) with decreasing expression frequency in MMCs (24.5%). Some suggested targets are not expressed (CD1B) or at a very low level in normal and malignant plasma cells (CD25 [2.6%]). Given are median expression in normal and malignant plasma cells, %age of patients expressing the respective gene, and standard deviation (SDV) within the respective population, i.e., BMPC or MMC. Note different expression height, e.g., detectable but low CD20 median expression. For graphical depiction of expression, see **Figure 1**. For expression in different MGUS and myeloma stages (asymptomatic myeloma, therapy-requiring myeloma, and relapsed myeloma), see **Supplementary Table S3**. For validation of target expression in the independent CoMMpass-cohort, see **Supplementary T** **able S4**.

Based on the expression pattern, we divided targets in those expressed in normal and almost all (>99%) malignant plasma cell samples (n=10; CD38, SLAMF7, BCMA, GPRC5D, FCRH5, TACI, CD74, CD44, CD37 and CD79B), those constitutively expressed in all normal plasma cells with expression loss in a subfraction of malignant plasma cells (n=4; BAFF-R [81.3%], CD19 [57.9%], CD20 [82.8%], CD22 [28.4%]), and targets aberrantly expressed in malignant plasma cells, i.e., not expressed in normal bone marrow plasma cells (n=3; NY-ESO1/2 [12%], MUC1 [12.7%], CD30 [4.9%]). CD70 is expressed in half of BMPC decreasing to 24.5% in MM. Of assessed targets, CD1B is not expressed, CD25 at very low frequency and expression height only (2.9%). Results for expressed targets are summarized in **Figures 1**, **4**, **Table 2** and **Supplementary Table S2**. All expressed targets show a significant variation in expression height (**Figure 1**, **Table 2**, **Supplementary Figure S1**, **Table S4** for validation in the independent CoMMpass cohort), as previously reported for CD38 (39), BCMA (58) and GPRC5D (43). See also **Table 2** (overview) and **Figure 1** for graphical depiction of expression in myeloma precursor entities and relapsed myeloma in comparison to normal plasma cells, normal proliferating plasmablastic cells, and HMCLs. Presence of the targetable BRAF V600E/K mutation was detected in 2.1% of patients in agreement with previous reports (29) and at a median allelic frequency (AF) setting in relation BRAF^wt^ vs. BRAF^mut^ of 60% (23% - 100%).

RNA-sequencing allows detection of somatic variants and mutated transcripts; e.g., for BCMA, we found in 25% of patients coding non-synonymous single nucleotide variants present (median 1, maximum 10) with a median allele frequency of 1 in patients harboring the aberration. If present, a resistance conveying mutation would thus have been detected but expectedly could not, as none of the patients has been treated with BCMA-targeting agents prior to analysis.

Targets expressed in all myeloma patients show high inter-patient variation, e.g., 6.8 (CD38), 6.9 (BCMA), 9.9 (GPRC5D), and 10.9 (FCRH5) log-fold variation (**Figure 1**, **Table 2**).

Immunological target expression decreases from MGUS to AMM to MM to MMR, except for the aberrantly expressed genes MUC1 and NY-ESO1.

### 3.2 Frequency of presence and height of expression

Frequency of presence of expression of the immune-oncological targets BAFFR, TACI, CD19, CD20, CD22 and CD37 significantly decreases, those for MUC1 and NY-ESO1 significantly increases from MGUS to AMM to MM to MMR as well as from AMM to MM to MMR. Presence of CD38, CS1, BCMA, GPRC5D, FCRH5, CD74, CD44, CD1B, CD30, CD70 and CD79B does not vary significantly in both comparisons, see **Supplementary T** **able S3**.

Of the ten targets constitutively expressed in normal plasma cells, i.e., CD38, SLAMF7 (CS1), BCMA, GPRC5D, FCRH5, TACI, CD74, CD44, CD37 and CD79B, seven show significant lower expression from MGUS to MMR (Jonckheere-Terpstra-Test, exploratory analysis), i.e., SLAMF7 (CS1), BCMA, GPRC5D, FCRH5, TACI, CD74, and CD37. If adjusted for multiple testing by Benjamini-Hochberg correction, significance is maintained for BCMA, FCRH5, TACI, CD74, and CD37. Targets with expression loss in a subfraction of malignant plasma cells of previously untreated patients (i.e., BAFF-R, CD19, CD20, CD22 [28.4%]) are all significantly lower expressed from MGUS to MMR (*P*-values adjusted for multiple testing). Of targets aberrantly expressed in malignant plasma cells, i.e., not expressed in BMPC (i.e., NY-ESO1/2, MUC1, CD30), NY-ESO1 shows significantly higher expression from MGUS to MMR. Regarding a potential use of immune-oncological agents in early-stage myeloma, we assessed differences between AMM to MM to MMR, (instead of MGUS-AMM-MM-MMR) yielding similar results (**Supplementary Figure S2**).

**Figure 5:**
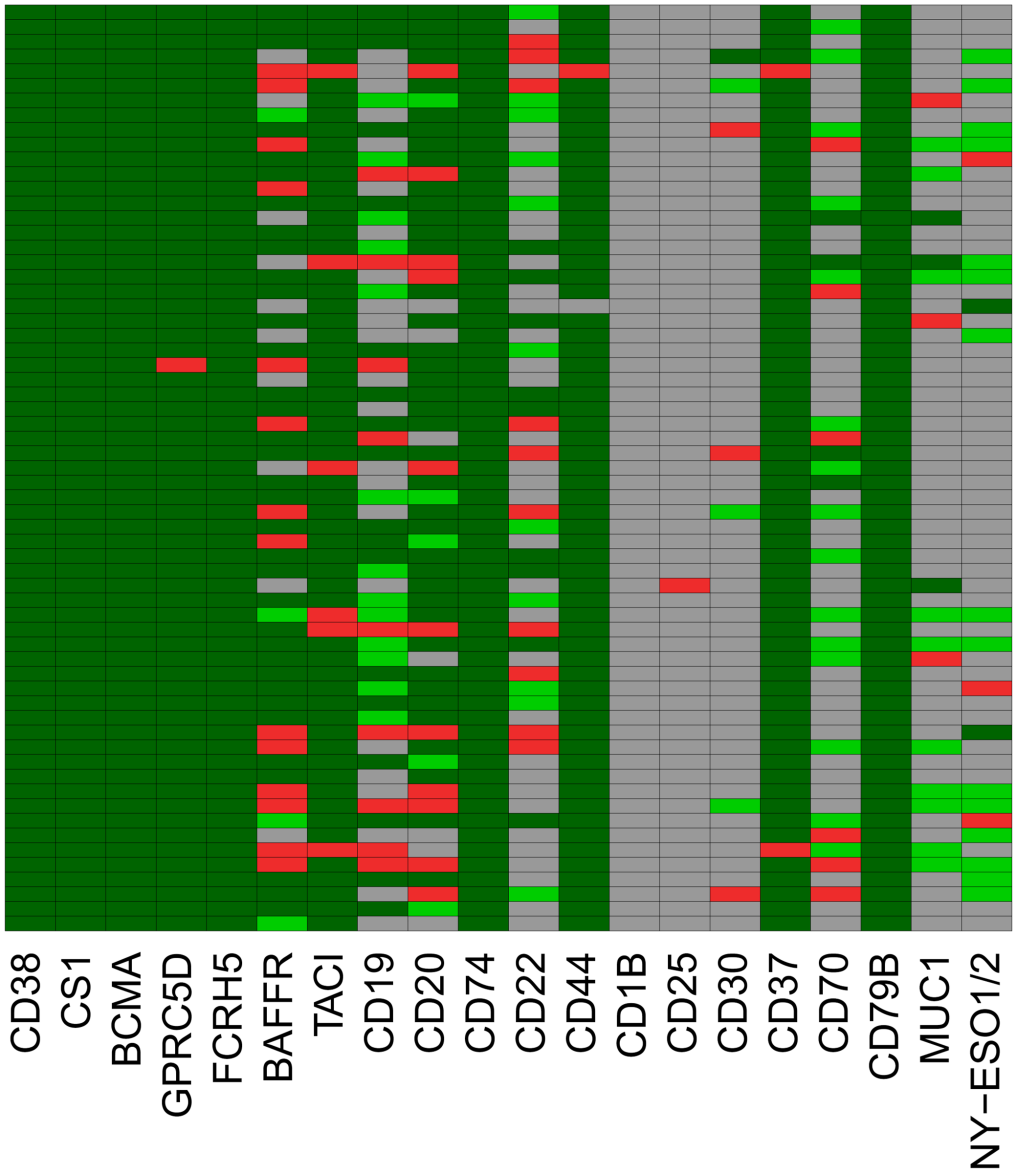
Targets expressed in previously untreated myeloma vary in expression in relapsed disease. Assessment in 63 patients. Each row depicts an individual patient assessed longitudinally at treatment initiation and relapse. Targets expressed in normal bone marrow plasma cells and multiple myeloma remain stable in longitudinal samples, especially if highly expressed (CD38, CS1, BCMA, FCRH5, CD74, CD79B). Genes for which expression is stage-dependently lost (e.g., CD19, CD22, BAFF-R) show high dynamics with changes occurring in 22/63 (35%), 21/63 (33%), 18/63 (19%) with comparable probability of gain and loss of expression. The cancer testis antigen expression regarding MUC1 [3 losses (red color) vs. 10 gains (light green color)] and NY-ESO1/2 and NY-ESO1/2 (2 losses vs. 15 gains) is predominantly gained in relapsed disease. Color code: dark green color, presence of expression in previously untreated myeloma and relapsed myeloma; light green, expression gained in relapsed myeloma; red – expression lost in relapsed myeloma; grey – no expression in both previously untreated myeloma and relapsed myeloma. See **Supplementary Tables S5, S6** for numerical depiction.

In longitudinal samples assessed at diagnosis and relapse (n=63), a change in expression of any of the investigated antigens could be found in 56 patients (88.9%) with both losses and gains occurring (**Figure 5**, **Supplementary Tables S5, S6**). Targets expressed in normal bone marrow plasma cells can be divided in those in which expression in myeloma cells remains stable in later stages in longitudinal samples (i.e., CD38, CS1, BCMA, FCRH5, CD74, CD79B), and those for which expression is stage-dependently lost (e.g., CD19, CD22, BAFF-R). The latter show high dynamics in progression from previously untreated to relapsed myeloma with changes occurring in 22/63 (35%), 21/63 (33%), and 18/63 (29%) of samples with comparable probability of gain and loss of expression. The cancer testis antigen expression regarding MUC1 (3 losses vs. 10 gains) and NY-ESO1/2 (2 losses vs. 15 gains) is predominantly gained in relapsed myeloma (**Figure 5**). Although the absolute differences in median expression are small (**Supplementary Table S6**), high downregulation can occur in individual patients. Significant (exploratory) downregulation in longitudinal patients is observed for GPRC5D, FCRH5, BAFFR, TACI, CD74, CD1B, CD25, CD30, CD37, as well as CD70, and upregulation for NY-ESO1/2 (**Supplementary** **Table S6**).

In a hypothetical scenario in which all treatment options as in **Table 1** are available, based on the expression pattern (**Figure 4**), for all patients a recommendation could be made.

### 3.3 Risk determination by RNA-sequencing

We first *de novo* generated a RNA-sequencing based score, termed LfM-HRS, using a method previously applied for DNA-microarrays (50). The LfM-HRS delineates three groups of patients with median EFS of 17 vs. 33 vs. 41 months (P<0.001) and OS of 33 vs. 83 vs. 143 months (P<0.001); **Figure 2**. For independent validation, we used the CoMMpass-cohort (**Supplementary Figure S2**) and translated the LfM-HRS into a DNA-microarray based score (**Supplementary Figure S3**). In both cases, it retained its prognostic significance.

To connect RNA-sequencing based risk assessment to gene expression-based risk scores by DNA-microarrays (“GEP”-scores), we “translated” these. The RS-, UAMS70- and SKY92-score delineated a population of 10%, 19%, and 13% of high risk and 67% of medium risk (Rs-score) patients. High- (medium) risk patients showed significantly inferior EFS and OS, i.e. the RS-score - RS^high^ vs. RS^medium^ vs. RS^low^ median EFS 17 vs. 33 vs. 43 months (P<0.001); median OS was 35 vs. 86 vs. 130 months (P<0.001, **Figure 2**); UAMS70 - median EFS of 21 vs. 36 months (P<0.001) and OS of 46 vs. 105 months (P<0.001); SKY92-signature median EFS of 15 vs. 35 months (P<0.001) and OS of 30 vs. 103 months (P<0.001).

GEP and RNA-sequencing based scores showed a concordance of 71.7% - 92.5% regarding patients identified as high risk (**Supplementary T** **able S7**).

### 3.4 Proliferation of malignant plasma cells

Proliferation of malignant plasma cells as biological variable is one of the strongest prognostic factors in myeloma (47, 65–68). We *de novo* generated a RNA-sequencing based proliferation index (RPI). In comparison to normal bone marrow plasma cells or non-proliferating memory B-cells, malignant plasma cells showed a significant and stage-dependent increase from early disease MGUS vs. asymptomatic vs. symptomatic, therapy-requiring multiple myeloma (**Figure 3A**; Jonckheere-Terpstra test P=0.001). Myeloma cell lines and plasmablasts showed a significantly higher proliferation rate. Patients with low/median/high RPI (34%/51%/15% of 535 MM) showed significantly different median EFS (39 vs. 31 vs. 24 months, P<0.001) and OS (128 vs. 82 vs. 51 months [P<0.001]; **Figure 3**). The RPI was validated on the CoMMpass-cohort (**Supplementary Figure S4**).

### 3.5 Comparison to iFISH and R-ISS based risk assessment

**Figure 6:**
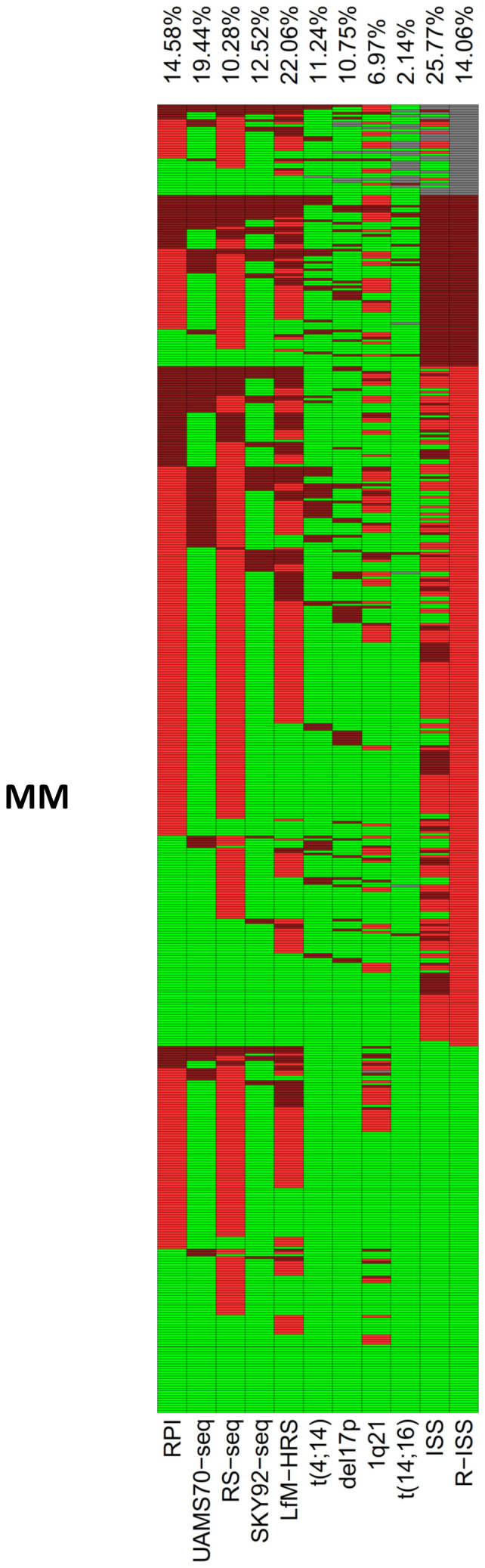
Determination of risk. Comparison of patients identified by RNA-sequencing scores, proliferation, R-ISS, and cytogenetic risk factors in 535 consecutive previously untreated myeloma patients. Percentage of patients identified as high risk and presence of t(4;14), del17p, 1q21 (>3 copies) or t(14;16) is depicted at the top of the figure and plotted in dark red color. Light red color delineates medium risk or presence of three copies of 1q21, green color low risk and/or absence of the respective aberrations. Grey color depicts missing values. Percentage of patients identified as high risk calculated excluding missing values.

Different patient populations are identified as high risk by iFISH, R-ISS and RNA-sequencing based assessment (**Figure 6**, **Supplementary Figures S5, S6**). iFISH identified 10.8% (57 of 530)/11.2% (60 of 534)/2.1% (11 of 514)/30.5% (126 of 531)/7.0% (37 of 531) of patients as harboring del17/t(4;14)/t(14;16)/ more or equal 3 copies/more than three copies of 1q21. Presence vs. absence of each aberration is associated with significantly adverse survival (data not shown). R-ISS identified 17.1% (70/498) of patients as high risk and was significantly predictive for EFS and OS (**Figure 2**, **Supplementary Figure S2**). Numerically, prediction of survival by RNA-sequencing was neither inferior to R-ISS (Brier-score, **Supplementary Figure S6**), nor better. However, R-ISS3 did neither identify a fraction of 30.51% (22/72) of patients with highly proliferative myeloma cells (RPI high) nor 82.8% (29/35) of patients with 1q21-gain (≥3 copies, not included in the R-ISS definition). Thus, by calculating R-ISS alone, part of high-risk patients is missed.

## 4 Discussion

### 4.1 Aplicability of RNA-sequencing in multicenter trials and clinical routine

We have previously shown GEP using DNA-microarrays to be possible in the GMMG-MM5 multicenter trial within four weeks in 81.9% of patients in which plasma cell purification was possible, and 75.8% of the total trial population (51). Here we show that using our small amount RNA-sequencing protocol (53) RNA-sequencing was possible in 92.5% of patients and 83.7% of the intention-to-treat population. Risk-scores summing over prognosis-associated genes as the LfM-HRS introduced here, those translated from GEP (UAMS70, SKY92, RS-score) and proliferation scores (RPI introduced here) delineated validated groups of patients with highly different EFS and OS. RNA-sequencing allows comparable although not statistically better prognostication (Brier-score) compared to standard of care R-ISS (46). The patient population identified as high risk differs to a certain degree between R-ISS vs. RNA-sequencing vs. individual chromosomal aberrations especially for highly proliferative myeloma patients and presence of more than three copies of 1q21, in agreement with previous reports (47–49, 52, 57, 69–72). R-ISS thus only identifies part of the high-risk population.

Considering potential immune-oncological targets, RNA-sequencing allows suggestions for all patients (IOnc-advisor). This first relates evidently to targets not expressed in all myeloma patients, as being either lost in a subfraction of malignant plasma cells, i.e., BAFF-R, CD19, CD20, and CD22, or gained, i.e., NY-ESO1/2, MUC1, and CD30. Secondly, it relates to high inter-patient variation of target expression. Considering targets expressed in all myeloma patients, 10.9 log-fold (FCRH5) differences were found. With reported relation of expression height and response for CD38 (43) and GPRC5D (41) and the general mechanisms of action of immune-oncological compounds, this likely applies to other targets as well. In case of two treatment options of comparable population-based response rates (assume BCMA- and FCRH5-CART) and lack of other deciding factors, odds of success could thus be potentially increased choosing the higher expressed target. Especially in patients having either limited reserves to tolerate rather aggressive treatment (e.g., CART), or limited coverage by health insurances, and considering that fewer patients receive subsequent lines of treatment (73), exemplified with 1^st^ (95%), to 2^nd^ (61%), 3^rd^ 38%, 4^th^(15%) and 5^th^ line (1%). Furthermore, use of γ-secretase inhibitors (e.g., crenigacestat) (44) in case of low BCMA-expression could be suggested. On a population basis, RNA-sequencing as presented here allows including myeloma patients with expressed rare targets in basket trials in other indications. The BRAF-V600E/K mutation, the best documented small molecule target in myeloma, was present in 2.1% of patients in our cohort, in agreement with previous reports (29). It exemplifies the identification of mutated transcripts by RNA-sequencing, easily extended to other targets once additional clinical evidence emerges.

A further emerging use of RNA-sequencing is assessment of antigen downregulation or loss under targeted treatment, as reported, e.g., for CD38 (43), GPRC5D (45, 74), or BCMA (45, 75). Both downregulation as well as coding mutations can be detected by RNA-sequencing as exemplified for BCMA. For GPRC5D, Mailankody et al. (74) showed for 6/6 patients progressing after CART (MCARH109) GPRC5D-downregulation (2/6) or loss of expression (4/6). Lee et al. (45) showed 4/6 patients progressing under GPRC5D-TCB to harbor biallelic mutations abrogating compound efficacy. Loss of BCMA-expression after anti-BCMA CART was initially reported as rare event (3/71; 4%) (75). Subsequent studies by Lee et al. (45) showed that in 8/16 investigated patients progressing under BCMA-directed treatment, biallelic deletions or mutations of the TNFRSF17 (BCMA) locus occurred: in two patients, MM relapse post T-cell bispecific antibody or CART-therapy was driven by BCMA-negative clones harboring focal biallelic deletions at the TNFRSF17 locus at relapse or by selective expansion of pre-existing subclones with biallelic TNFRSF17 loss. In further five relapsing patients, newly detected non-truncating, missense mutations, or in-frame deletions in the extracellular domain of BCMA negated the efficacies of anti-BCMA T-cell bispecific antibody therapies, despite detectable surface BCMA protein expression. Of specific interest, for four BCMA mutational events, distinct sensitivities toward different anti-BCMA-targeting therapies could be found: first, a p.Arg27Pro mutation conferred resistance against teclistamab and elrantamab, abrogating binding and activity. Here, BCMA Arg27 interacts with the complementarity-determining regions of the heavy chain of the anti-BCMA variable region of teclistamab. In contrast, binding and activity of alnuctamab or Ide-cel-analogous CART is maintained. Secondly, a p.Pro34del in-frame deletions conveyed resistance against teclistamab and elrantamab but maintained alnuctamab binding and activity, and third, a p.Ser30del conveyed to teclistamab but retained sensitivity to elranatamab and alnuctamab (45). As relapse under one BCMA-targeting agent does therefore not necessarily implicate resistance against others, and RNA-sequencing can easily identify these mutations, it can be used to guide subsequent treatment lines with *different* BCMA-targeting agents. As downregulations and mutations discussed here occur primarily under selection pressure of the respective treatment and, in part, different aberrations occur in a subclonal manner (45), with the lack of this evolutionary pressure, and on the background of clonal heterogeneity of myeloma, other subclones can grow out after subsequent lines of treatment, conveying, e.g., again substantial expression of CD38 or GPRC5D, which can, in the same way, be identified by RNA-sequencing.

### 4.2 Longitudinal samples

Longitudinal samples showed dynamic changes of expression between diagnosis and relapse in 88.9% of patients for any of the investigated antigens, with both losses and gains occurring. These changes refer less to those targets likewise expressed in normal bone marrow plasma cells, especially those highly expressed (CD38, CS1, BCMA, FCRH5, CD74, CD79B). Genes with stage-dependent loss of expression, like CD19, CD22, and BAFF-R, showed high dynamics with both gains and losses of expression occurring. The cancer testis antigens MUC1 and NY-ESO1/2 were predominantly gained in MMR. These findings are in line with a subclonal architecture in myeloma leading to clonal tides (76) and spatio-temporal evolution between diagnosis and relapse (77). Molecular assessment should thus be repeated in relapse, especially for targets expressed with high dynamics, or if specific treatment has been applied, as e.g., CD38 or BCMA-directed therapy, leading to a selection pressure regarding target downregulation or loss.

### 4.3 Implementation of RNA-sequencing in standard work-up of myeloma patients

Introduced in myeloma research in 2002 (78) and 2011 (79), GEP and NGS revolutionized our understanding of myeloma biology, pathogenesis, and risk (80, 81) but the standard myeloma-workup is still based on morphological bone marrow assessment and iFISH. Why is this the case?

Several reasons can be identified. In particular, a knowledge gap between routine clinical care and the field of molecular profiling: GEP (23) and NGS (80) can be perceived as slow, complex, expensive, and not broadly applicable techniques that return results hard to interpret and reproduce, and with little clinical value. Of these, “practical issues” can be easily disproven: GEP can be applied in clinical routine in academic [e.g., GEP-R (82), UAMS70-score (48), IFM-score (49)] and commercial settings [e.g., MyPRS^®^, Signal Genetics™ (83), MMprofiler™, SkylineDiagnostics (72)] in most patients (51) within four weeks (51). NGS-based techniques, e.g., for mutational profiling or sequencing based FISH, can be performed in academic (CoMMpass) (84) or routine private laboratory setting (85), even within 14 days in a tertiary hospital (86). RNA-sequencing can be used in academic (39, 58, 84) or private laboratory settings (87) in over 90% of patients in clinical trials or routine within four weeks. But for rare circumstances, myeloma treatment is not an emergency, and a time interval of four weeks can be covered with a short course of steroids while waiting for test results (80). GEP or NGS-based sequencing are not expensive: cost in the range of 1000 US$ are comparable to iFISH (depending on the number of probe sets used) and frequently less than ten-fold compared with monthly treatment costs.

Are results then “hard to interpret and reproduce” and of “little clinical value”? Clinical value is given by risk assessment for patient counselling and respective trial-inclusion and targets selection for individualized treatment in a context of multiple equal-seeming options.

But first, there is no consensus as to whether, and how, use these techniques to re-define risk. For GEP, a variety of prognostic scores (48, 49, 82) identifies partially overlapping patient populations and depend, to an extent, on the applied methodology (e.g., Affymetrix single vs. double amplification protocol). RNA-sequencing can apply translated GEP-based- or *de novo* generated scores (88) and sequencing-based R-ISS can be used (84). Mutational signatures can be prognostic (89, 90). Even if numerically superior, at the end of the day, GEP or NGS-based risk assessment are to be perceived, at large, as not better than R-ISS, not standardized and thus not warranted in routine application. This might change with NGS-based re-defining of adverse risk factors like the t(4;14) translocation depending on the breakpoint within the NSD2 gene (91). Secondly, although “risk” is part of treatment decisions, e.g., in the Mayo clinic’s mSMART-stratification (www.msmart.org) (92), suggesting e.g., bortezomib maintenance for patients harboring t(4;14), del17p, t(14;20), t(14;16) (or transplant eligible additionally 1q-gain and double/triple hit multiple myeloma), the GMMG suggesting bortezomib maintenance after HDT and ASCT for previously untreated myeloma patients harboring a del17p13 or t(4;14), the GMMG-CONCEPT-trial (iFISH-based), the UAMS total therapy program (GEP-based) (93) or the MUKine-OPTIMUM trial (GEP/iFISH-based) (72). But there is still hesitation to apply risk-based approaches, because although low risk-patients might be spared “unnecessarily more effective” treatment and costs saved, for no treatment it is currently shown to works *better* in high risk compared to low-risk patients, and thus would be applicable for all patients.

Immediate usefulness would be perceived if either response could be predicted, or targets selected in for personalized treatment. Despite factors associated with response have been identified (24, 25), it has not been possible to predict response to non-targeted small molecules at a clinically applicable level (22, 23). In contrast, targetable mutations can be identified as exemplified by the BRAF V600E/K mutation (vemurafenib) (29), present in 2.1% of patients in our cohort, in agreement with previous reports (29). Currently, several clinical trials, e.g., “A Study to Evaluate Myeloma-Developing Regimens Using Genomics (MyDRUG, NCT02884102)” for patients with ≥30% mutation of CDKN2C, FGFR3, KRAS, NRAS, BRAF V600E, IDH2, or translocation t(11;14), and “Targeted Therapy Directed by Genetic Testing in Treating Patients With Advanced Refractory Solid Tumors, Lymphomas, or Multiple Myeloma (The MATCH Screening Trial, NCT02465060)” address this question. Although in principle GEP would have been applicable for target selection, attempts failed due to lack of compounds usable for personalized treatment, and suggested compounds like inhibitors of aurora kinase (57, 82) or IGF1R (82, 94) never made it to approval in myeloma. The situation is very different now with compounds available for personalized treatment both targeting immune-oncological targets and mutations which can be identified by NGS-techniques as RNA-sequencing. In the latter case, comprising change of BCMA-targeting treatment to, e.g., different T-cell bispecific antibodies in case of specific mutations. Clinical usefulness is thus now *a priori* evident.

GEP/NGS-based approaches would be significantly fostered by the use of appropriated clinical trial designs, especially for regulatory and approval purposes (81, 95) especially when considering the number of compounds and combinations. Traditional designs like phase III randomization of NGS (RNA-sequencing) guided vs. investigators choice will be very difficult to implement, as, based on lack of governmental funding, pharmaceutical companies would need to be willing to provide their compounds without necessarily aligning business interests, which is, based on experience in IIT-trial design, very unlikely. NGS-based approaches are, however, implemented as part of tumor boards (96) as institutionalized framework for decision making and consecutively enabling refunding of treatments outside their specific indication by health insurances. “Educated first choice” of treatment in case of lack of other guidance as part of personalized risk benefit assessment as suggested here is a further complementary possibility. Either way, precision oncology represents an epochal revolution in patients’ management, and therefore it is conceivable that it involves substantial changes (at both cultural and practical levels) in the way we operate in order to cure cancer, that surely will need a long time to be realized. The concerted effort of all stakeholders involved in the development of precision oncology (researchers, clinicians, regulatory agencies, governments) is now mandatory to ensure that in the future it will become a reality in routine clinical practice (81).

## 5 Conclusion

RNA-sequencing is applicable in 90% of patients (comparable to iFISH), is of overall equal predictive power as the current gold standard R-ISS, but identifies a further fraction of myeloma patients, e.g., with highly proliferative myeloma cells. It allows personalized target identification for immune-oncological drugs based on presence and height of expression. RNA-sequencing used for “educated first guess” could be considered as “IOnc drug and risk advisor” in analogy to other decision tools.

## Data availability statement

The datasets presented in this study can be found in online repositories. The names of the repository/repositories and accession number(s) can be found below: https://www.ebi.ac.uk/arrayexpress/, E-MTAB-4715; https://www.ebi.ac.uk/arrayexpress/, E-MTAB-4717; https://www.ebi.ac.uk/arrayexpress/, E-MTAB-5212; https://www.ebi.ac.uk/arrayexpress/, E-TABM-937; https://www.ebi.ac.uk/arrayexpress/, E-TABM-1088; https://www.ebi.ac.uk/ena, PRJEB37100; https://www.ebi.ac.uk/ena, PRJEB36223.

## Ethics statement

Patients were included in the study approved by the ethics committee of the medical faculty of the Ruprecht Karls University Heidelberg, Germany, (#229/2003, #S-152/2010) after written informed consent. The studies were conducted in accordance with the local legislation and institutional requirements. The participants provided their written informed consent to participate in this study.

## Author contributions

ME-R: Conceptualization, Formal Analysis, Investigation, Methodology, Validation, Writing – review & editing. SB: Formal Analysis, Writing – review & editing. VB: Writing – review & editing, Investigation, Methodology. HS: Investigation, Writing – review & editing. UB: Investigation, Writing – review & editing. CS: Investigation, Writing – review & editing. MH: Investigation, Writing – review & editing. KW: Investigation, Writing – review & editing. TH: Investigation, Writing – review & editing, Methodology. MR: Investigation, Writing – review & editing. HG: Investigation, Writing – review & editing, Funding acquisition. AJ: Investigation, Writing – review & editing. KM: Investigation, Writing – review & editing. EB: Investigation, Writing – review & editing. EM: Investigation, Writing – review & editing. KD: Investigation, Writing – review & editing. JM: Investigation, Writing – review & editing. KV: Investigation, Writing – review & editing. AS: Conceptualization, Formal Analysis, Funding acquisition, Investigation, Supervision, Writing – original draft. DH: Conceptualization, Formal Analysis, Funding acquisition, Investigation, Methodology, Supervision, Validation, Writing – original draft.
