# Supplementary material for: RNA-sequencing based first choice of treatment and determination of risk in multiple myeloma
Source: Front Immunol. 2023 Nov 15;14:1286700. doi: 10.3389/fimmu.2023.1286700 (PMC10684778; doi:10.3389/fimmu.2023.1286700)
Supplement: Supplementary file 1 [file DataSheet_1.pdf]

SUPPLEMENTARY APPENDIX

SUPPLEMENTARY DATA 2

SUPPLEMENTARY FIGURES 2

SUPPLEMENTARY TABLES 11

SUPPLEMENTARY METHODS 17

REFERENCES 21

SUPPLEMENTARY DATA

SUPPLEMENTARY FIGURES

Supplementary Figure S1. RNA-sequencing assisted educated first choice of immune-oncological actionable targets (IOnc-advisor). Independent validation on a cohort of 767 symptomatic previously untreated myeloma patients (CoMMpass-cohort). Grey color indicates absence of target expression. Green color indicates expression. Percentage of expression is depicted at the top of the figure. Target overexpression in comparison to the median expression within the myeloma cell population ± one standard-deviations is depicted in light (lower expression) and dark (higher expression) green.

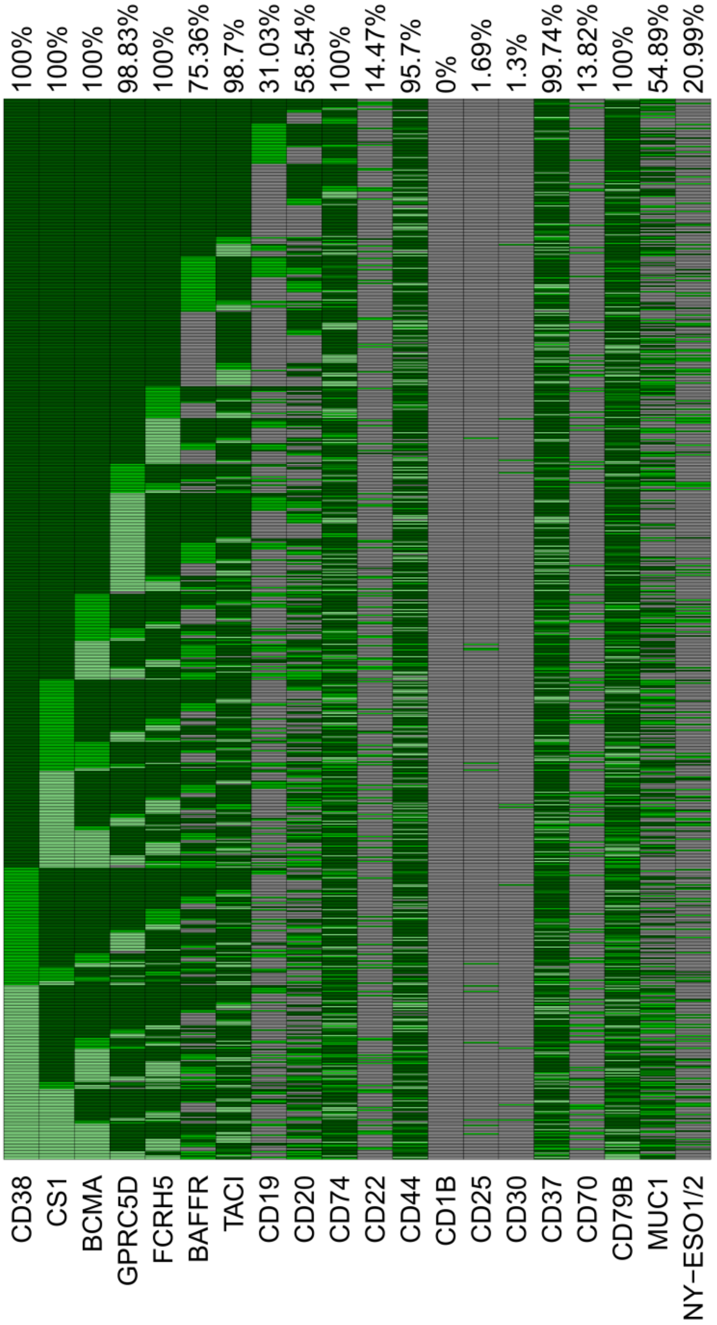

Supplementary Figure S2. **RNA-sequencing based assessment of immune-oncological actionable targets** depicted in Figure 1. Expression height in malignant plasma cells from MGUS-, asymptomatic (AMM), symptomatic (MM) and relapsed myeloma patients (MMR) in comparison to normal bone marrow plasma cells (BMPC), memory B-cells (MBC), proliferating plasmablasts (PPC) and human myeloma cell lines (HMCL). Targets can be divided in those expressed in all normal and (almost all, >99%) malignant plasma cells (n=10; CD38, SLAMF7 (CS1), BCMA, GPRC5D, FCRH5, TACI, CD74, CD44, CD37 and CD79B), those constitutively expressed in all normal plasma cells with expression lost in a subfraction of malignant plasma cells (n=4; BAFF-R [81.3%], CD19 [57.9%], CD20 [82.8%], CD22 [28.4%]), and targets aberrantly expressed in malignant plasma cells, i.e., not expressed in BMPC, (n=3; NY-ESO1/2 [12%], MUC1 [12.7%], CD30 [4.9%]). Some suggested targets are not expressed (CD1B) or at a very low level in normal and malignant plasma cells (CD25 [2.6%]). Black and grey color of data points and corresponding numbers indicate "presence" and "absence" of expression, respectively. See Table 2 for numerical depiction and details. "Present" expression by RNA-sequencing is defined as presence of at least one read count per million (CPM) per 1000 bp. Gene length is defined as median transcript length. Significant difference for higher (all other genes)- or lower expression (MUC1, NY-ESO1) of genes from MGUS to AMM to MM to MMR is assessed by Jonckheere-Terpstra Test (JHT). Exploratory *P*-values given. P-Values remaining significant after Benjamini-Hochberg adjustment for multiple testing are depicted in blue color. Note: part of expression data for BCMA and CD38 have previously been published (Seckinger et. al., 2017 and Seckinger et al., 2018). For a comparison from MGUS to AMM to MM to MMR, see Figure 2.

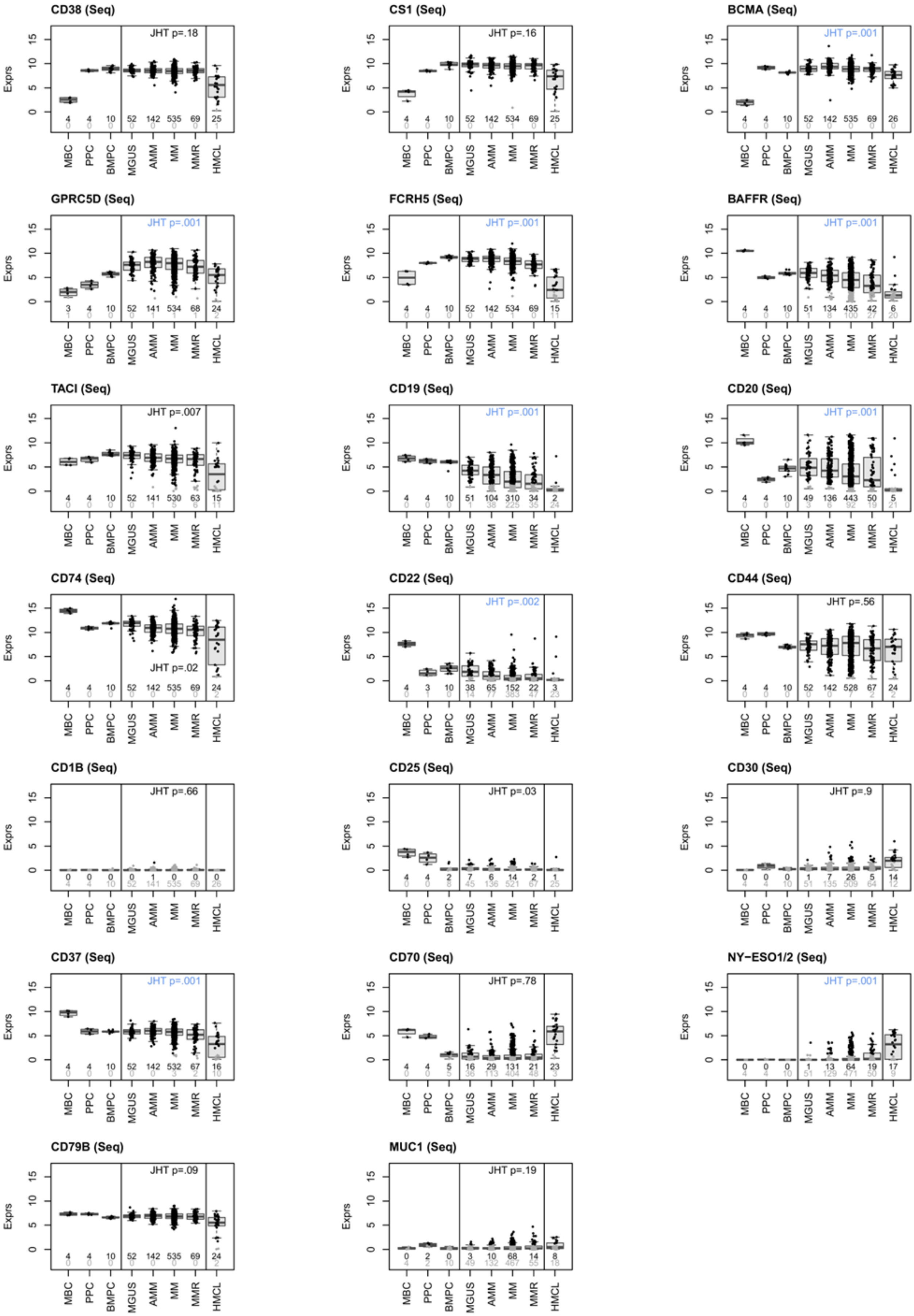
CD38 (Seq)
JHT p=.18
CS1 (Seq)
JHT p=.16
BCMA (Seq)
JHT p=.001
GPRC5D (Seq)
JHT p=.001
FCRH5 (Seq)
JHT p=.001
BAFFR (Seq)
JHT p=.001
TACI (Seq)
JHT p=.007
CD19 (Seq)
JHT p=.001
CD20 (Seq)
JHT p=.001
CD74 (Seq)
JHT p=.02
CD22 (Seq)
JHT p=.002
CD44 (Seq)
JHT p=.56
CD1B (Seq)
JHT p=.66
CD25 (Seq)
JHT p=.03
CD30 (Seq)
JHT p=.9
CD37 (Seq)
JHT p=.001
CD70 (Seq)
JHT p=.78
NY−ESO1/2 (Seq)
JHT p=.001
CD79B (Seq)
JHT p=.09
MUC1 (Seq)
JHT p=.19
Exprs
MBC
PPC
BMPC
MGUS
AMM
MM
MMR
HMCL

Supplementary Figure S3. RNA-sequencing based determination of risk. Independent validation on a cohort of 767 symptomatic previously untreated myeloma patients (CoMMpass-cohort). **A**. De novo generated RNA-sequencing-based scores for risk (LfM-HRS, **A1**) and proliferation (RPI, **A2**) delineates three groups with significantly different event-free (EFS) and overall survival (OS). **B. "GEP"-scores** translated into RNA-sequencing. The scores of the Universities of Heidelberg and Montpellier (RS-score) (**B1**), the University of Arkansas Medical School (UAMS70) (**B2**), and the Erasmus Medical Center (SKY92), (**B3**) in each case delineate symptomatic myeloma patients with significantly different EFS and OS. **C**. The current clinical gold standard (revised ISS-score) delineates three groups of 24%, 64% and 12% of 767 patients with significantly different OS (**C1**) and EFS (**C2**). Depicted are Kaplan Maier curves with log-rank based *P*-value and patients at risk. *P*-values were adjusted for multiple testing using Benjamini-Hochberg correction.

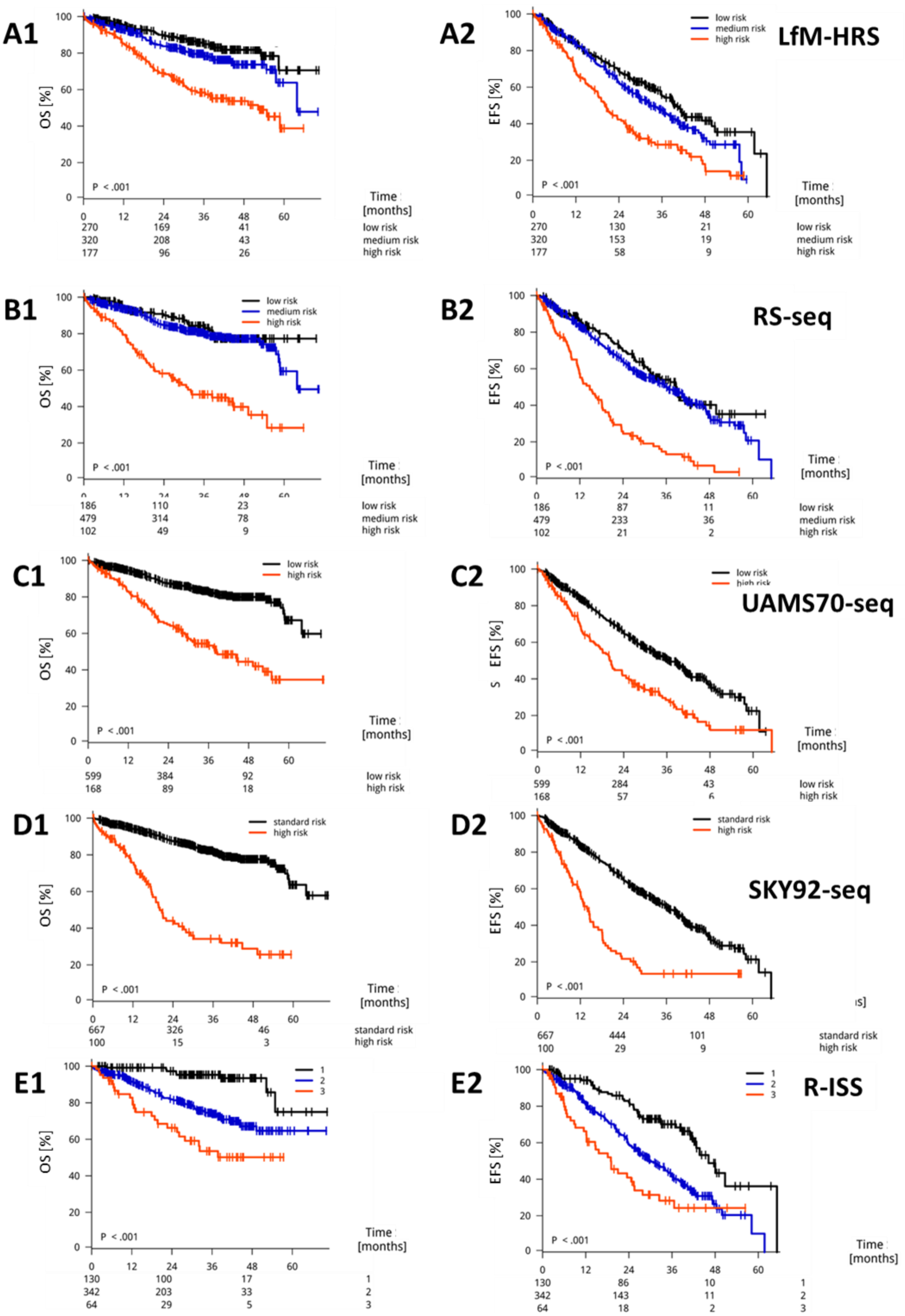
A1
OS [%]
P < .001
Time [months]
0 12 24 36 48 60
low risk 270 169 41
medium risk 320 208 43
high risk 177 96 26
A2
LfM-HRS
low risk
medium risk
high risk
EFS [%]
P < .001
Time [months]
0 12 24 36 48 60
low risk 270 130 21
medium risk 320 153 19
high risk 177 58 9
B1
low risk
medium risk
high risk
OS [%]
P < .001
Time [months]
low risk 186 110 23
medium risk 479 314 78
high risk 102 49 9
B2
RS-seq
EFS [%]
P < .001
Time [months]
0 12 24 36 48 60
low risk 186 87 11
medium risk 479 233 36
high risk 102 21 2
C1
low risk
high risk
OS [%]
P < .001
Time [months]
0 12 24 36 48 60
low risk 599 384 92
high risk 168 89 18
C2
UAMS70-seq
low risk
EFS [%]
P < .001
Time [months]
0 12 24 36 48 60
low risk 599 284 43
high risk 168 57
D1
standard risk
high risk
OS [%]
P < .001
Time [months]
0 12 24 36 48 60
standard risk 667 326 46
high risk 100 15 3
D2
SKY92-seq
standard risk
high risk
EFS [%]
P < .001
0 12 24 36 48 60
standard risk 667 444 101
high risk 100 29 9
E1
1
2
3
OS [%]
P < .001
Time [months]
0 12 24 36 48 60
1 130 100 17
2 342 203 33
3 64 29 5
E2
R-ISS
1
2
3
EFS [%]
P < .001
Time [months]
0 12 24 36 48 60
1 130 86 10
2 342 143 11
3 64 18 2

Supplementary Figure S4. **Translation of RNA-sequencing based determination of risk (LfM-HRS) into GEP-based** leads to delineation of three groups of patients with significantly different survival. **A.** Event-free survival (EFS), **B.** overall survival (OS). Cohort of 535 symptomatic previously untreated myeloma patients. Depicted are Kaplan Maier curves with log-rank based *P*-value and patients at risk. *P*-values were adjusted for multiple testing using Benjamini-Hochberg correction.

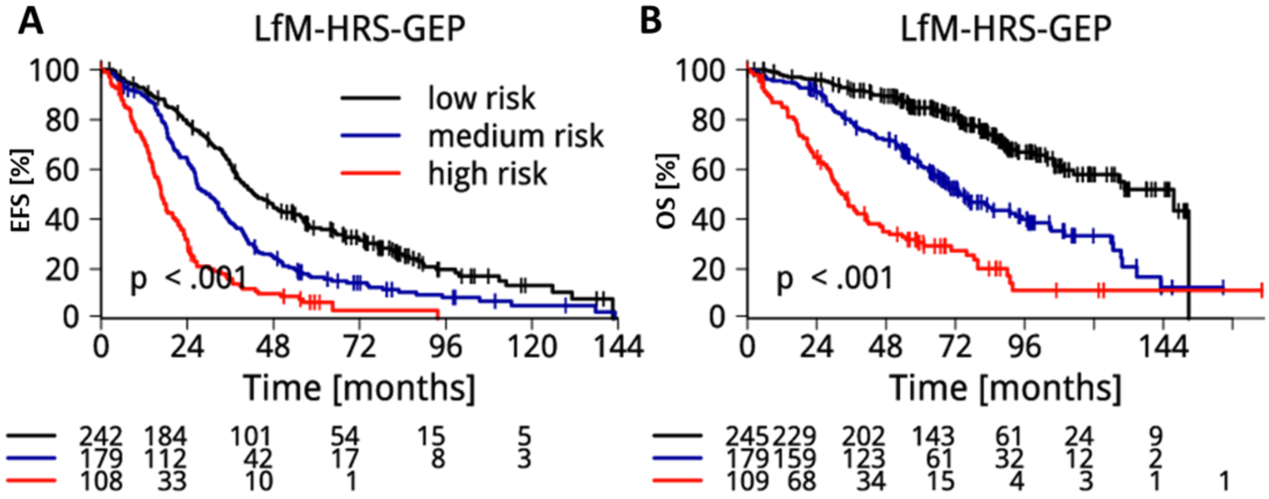

<u>Supplementary Figure S5</u>. <u>RNA-sequencing based determination of proliferation</u> (RPI). Independent validation on a cohort of 767 symptomatic previously untreated myeloma patients (CoMMpass-cohort). Significant differences are depicted by asterisks (*** P<.001, ** P<0.01, * P<0.05). **<u>A</u>.** Event-free survival (EFS), **<u>B</u>.** Overall survival (OS). Depicted are Kaplan Maier curves with log-rank based *P*-value and patients at risk. *P*-values were adjusted for multiple testing using Benjamini-Hochberg correction.

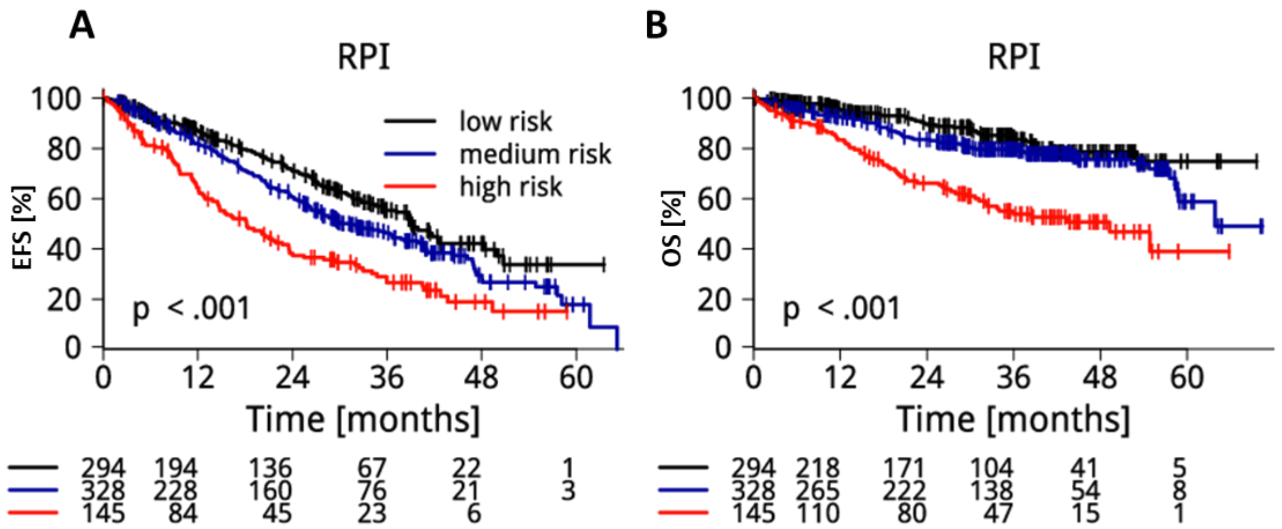

Supplementary Figure S6. Determination of risk. Comparison of patients identified by RNA-sequencing scores, proliferation, R-ISS and cytogentic risk factors. Independent validation on a cohort of 767 symptomatic previously untreated myeloma patients (CoMMpass-cohort). Percentage of high risk and presence of t(4;14), del17p, 1q21 and t(14;16) is depicted at the top of the figure and plotted in dark red color. Light red color delineates medium risk, green color low risk and/or absence of the respective aberrations. Grey color depicts missing values. Percentage of patients as identified as high risk calculated excluding missing values. For comparison (LfM-cohort), see Figure 6.

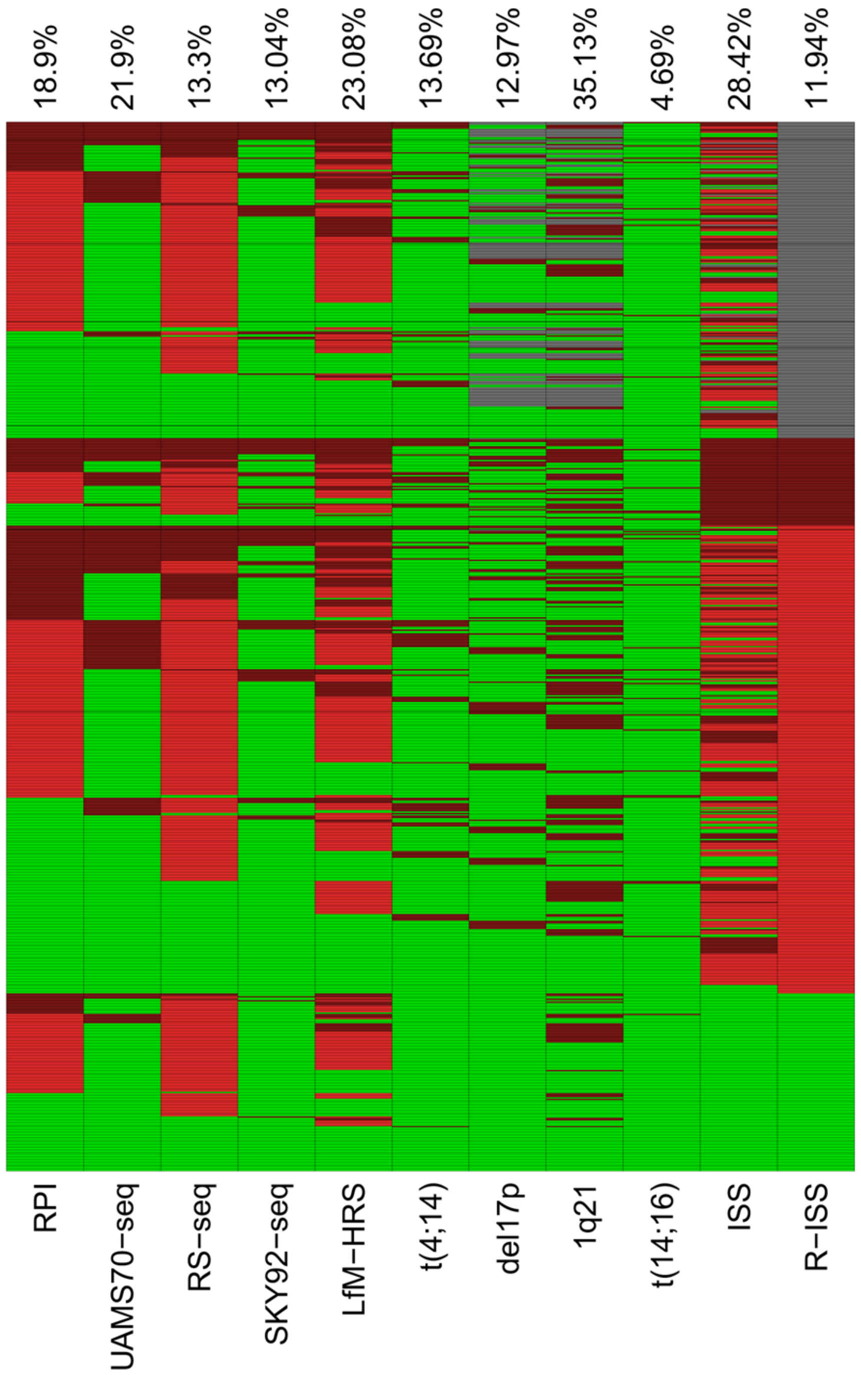

Supplementary Figure S7. **Brier-Score** comparing different RNA-sequencing based risk-assessments against the R-ISS score. Reference: Kaplan-Meier prediction model. A. LfM cohort, B. CoMMpass-cohort.

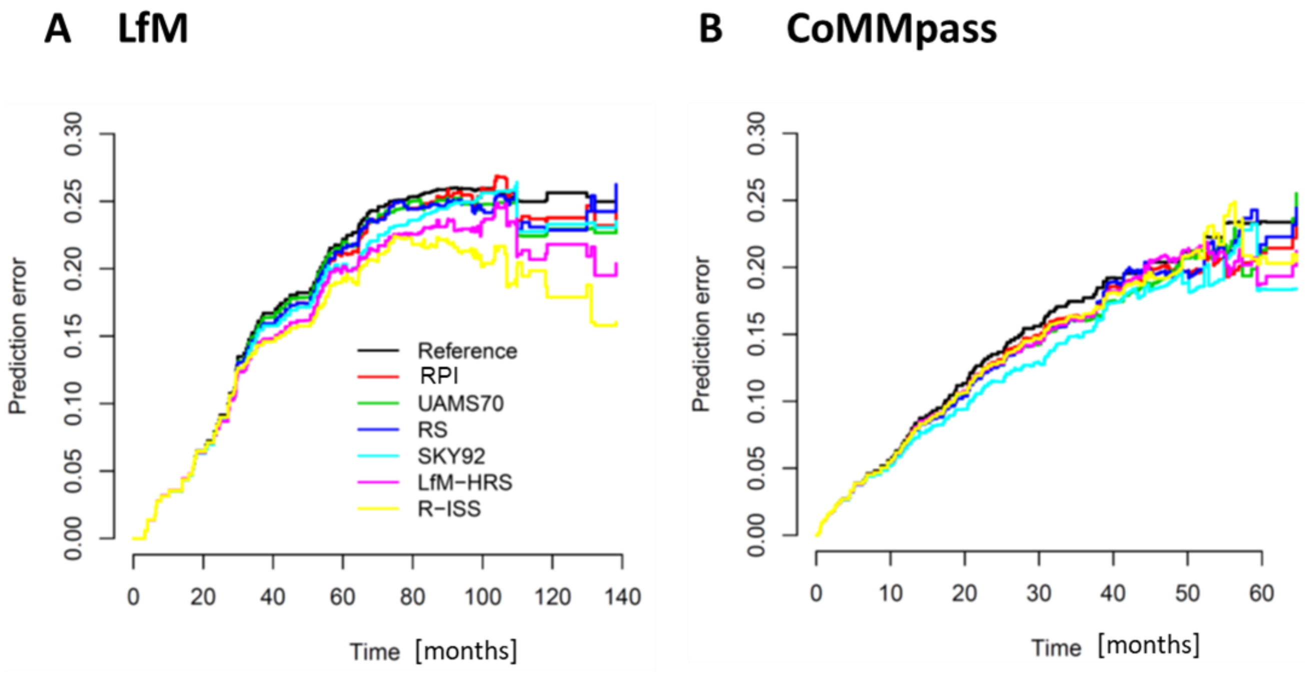

SUPPLEMENTARY TABLES

Supplementary Table S1. Patient characteristics and cohorts.

Variable	Level	MGUS		AMM		MM		MMR	
		n	%	n	%	n	%	n	%
Sex	Male	28	53,8	69	48,6	218	40,7	27	39,1
	Female	24	46,2	73	51,4	317	59,3	42	60,9
Age [years]	≤60	21	40,4	65	45,8	287	53,6	24	34,8
	>60	31	59,6	77	54,2	248	46,4	45	65,2
	NA								
Type	IgA	11	21,2	33	23,2	101	18,9	8	11,6
	IgG	40	76,9	98	69	319	59,6	46	66,7
	IgD	0	0	0	0	3	0,6	0	0
	Bence Jones	0	0	10	7	104	19,4	12	17,4
	Double gamn	1	1,9	0	0	0	0	0	0
	Asecretory	0	0	0	0	4	0,7	3	4,3
	Other	0	0	1	0,7	4	0,8	0	0
Light chain type	Kappa	28	53,8	85	59,9	353	66	46	66,7
	Lambda	24	46,2	57	40,1	177	33,1	20	29
	Asecretory	0	0	0	0	4	0,7	3	4,3
	NA	0	0	0	0	1	0,2	0	0
Plasma cell infiltration [%]	<10	45	86,5	14	9,9	35	6,5	10	14,5
	≥10	6	11,5	85	59,9	110	20,6	21	30,4
	≥30	0	0	28	19,7	169	31,6	15	21,7
	≥60	0	0	7	4,9	160	29,9	11	15,9
	NA	1	1,9	8	5,6	61	11,4	12	17,4
Monoclonal protein [g/l]	<20	48	92,3	59	41,5	102	19,1	24	34,8
	≥20	4	7,7	34	23,9	67	12,5	13	18,8
	≥30	0	0	41	28,9	280	52,3	11	15,9
	NA	0	0	8	5,6	86	16,1	21	30,4
Urinary monoclonal protein [mg/24h]	<500	49	94,2	117	82,4	286	53,5	40	58
	≥500	1	1,9	14	9,9	158	29,5	13	18,8
	NA	2	3,8	11	7,7	91	17	16	23,2
R-ISS stage	1	29	55,8	77	54,2	150	28	16	23,2
	2	17	32,7	41	28,9	278	52	23	33,3
	3	1	1,9	4	2,8	70	13,1	2	2,9
	NA	5	9,6	20	14,1	37	6,9	28	40,6

MGUS, monoclonal gammopathy of unknown significance; AMM, asymptomatic myeloma; MM, symptomatic, therapy-requiring multiple myeloma; MMR, relapsed multiple myeloma; Ig, immunoglobulin; R-ISS, revised International Staging System.

Supplementary Table S2. RNA-sequencing based assessment of immune-oncological actionable targets (IOnc-advisor) depicted for normal bone marrow plasma cells (BMPC), multiple myeloma cells (MMC) from MGUS, AMM, therapy requiring MM (MM), relapsed MM (MMR), and as controls, memory B-cells (MBC), in vitro differentiated plasmablastic cells (PPC), and human myeloma cell lines (HMCL). For summary depiction of MM, see Table 2. SDV, standard deviation.

TARGET	Expression BMPC						Expression MMC (MGUS)						Expression MMC (AMM)						Expression MMC (MM)						Expression MMC (MMR)					
	n	%	median	min	max	SDV	n	%	median	min	max	SDV	n	%	median	min	max	SDV	n	%	median	min	max	SDV	n	%	median	min	max	SDV
CD38	10	100	9	8,1	9,6	0,48	52	100	8,6	7,3	10	0,53	142	100	8,6	5,5	10,5	0,71	535	100	8,5	4,1	10,9	0,83	69	100	8,6	6,5	10,3	0,76
CS1	10	100	9,8	8,8	10,3	0,47	52	100	9,8	4,5	11,7	1,12	142	100	9,7	5,4	11,2	0,9	534	99,8	9,5	0,9	11,7	0,85	69	100	9,7	6,6	11	0,87
BCMA	10	100	8,2	7,8	8,6	0,22	52	100	9	7,8	10,8	0,76	142	100	9,4	2,4	13,6	1,03	535	100	8,9	4,8	11,7	0,9	69	100	9	6,3	11,7	0,85
GPRC5D	10	100	5,8	5,1	6,2	0,37	52	100	7,6	4,3	10,3	1,25	141	99,3	8,2	0,7	10,6	1,75	534	99,8	8	1	10,9	1,74	68	98,6	7,2	0,7	10,6	1,87
FCRH5	10	100	9,1	8,5	9,6	0,29	52	100	8,9	7,4	10,4	0,73	142	100	9	2,6	10,8	1,11	534	99,8	8,4	1,1	12	1,12	69	100	7,7	3,2	9,8	1,24
BAFFR	10	100	5,8	5,5	6,6	0,39	51	98,1	5,9	0,9	8,1	1,46	134	94,4	5,4	0,5	8,9	1,69	435	81,3	4,5	0	9,1	2,12	42	60,9	3,3	0,1	8,6	2,35
TACI	10	100	7,6	7,3	8,5	0,41	52	100	7,4	2,7	9,3	1,24	141	99,3	6,9	0,8	9,6	1,29	530	99,1	6,7	0	13,1	1,49	63	91,3	6,7	0,2	8,8	2,16
CD19	10	100	6,1	4,1	6,3	0,66	51	98,1	4,2	0,8	7	1,41	104	73,2	3,3	0	8	2,06	310	57,9	2	0	9,6	2,2	34	49,3	1,5	0	7,8	1,99
CD20	10	100	4,7	3	6,5	1,02	49	94,2	4,8	0,5	11,6	2,57	136	95,8	4,2	0,5	11,3	2,64	443	82,8	3	0	11,8	3,02	50	72,5	2,3	0	11	3,48
CD74	10	100	11,9	10,8	12,2	0,38	52	100	11,9	8,3	13,4	1,06	142	100	10,9	6,1	13,3	1,24	535	100	10,8	5,8	16,9	1,52	69	100	10,5	5,7	13,3	1,62
CD22	10	100	2,6	1,5	3,6	0,73	38	73,1	1,8	0	5,7	1,26	65	45,8	0,9	0	4,1	1,04	152	28,4	0,5	0	9,5	0,92	22	31,9	0,5	0	8,7	1,3
CD44	10	100	7	6,6	7,5	0,29	52	100	7,5	2,9	9,8	1,54	142	100	7,3	1,1	10,5	2,23	528	98,7	7,8	0,5	11,8	2,59	67	97,1	6,7	0,4	11,3	2,67
CD1B	0	0	0	0	0,4	0,11	0	0	0	0	0,9	0,16	1	0,7	0	0	1,6	0,19	0	0	0	0	1,1	0,14	0	0	0	0	1,1	0,17
CD25	2	20	0,1	0	1,7	0,62	7	13,5	0,2	0	2,1	0,48	6	4,2	0,1	0	2,4	0,39	14	2,6	0,1	0	2,3	0,33	2	2,9	0,1	0	1,4	0,29
CD30	0	0	0,2	0	0,6	0,19	1	1,9	0,2	0	2,1	0,4	7	4,9	0,2	0	4,8	0,73	26	4,9	0,2	0	5,8	0,67	5	7,2	0,4	0	2,9	0,66
CD37	10	100	5,8	5,4	6,3	0,23	52	100	5,9	4,1	8,1	0,77	142	100	6	2,8	8	0,87	532	99,4	5,8	0,7	8,4	1,1	67	97,1	5,2	0,3	7,4	1,56
CD70	5	50	1,1	0,2	1,6	0,47	16	30,8	0,6	0	6,3	1,12	29	20,4	0,4	0	5,8	0,94	131	24,5	0,3	0	7,5	1,2	21	30,4	0,5	0	5,9	1,12
CD79B	10	100	6,6	6,3	6,9	0,15	52	100	6,9	6	8,7	0,47	142	100	6,9	5,2	8,4	0,66	535	100	6,8	4,2	9	0,72	69	100	6,8	5,4	8,3	0,69
MUC1	0	0	0,2	0	0,6	0,22	3	5,8	0,2	0	1,6	0,35	10	7	0,2	0	2,2	0,41	68	12,7	0,2	0	3,6	0,54	14	20,3	0,2	0	4,7	0,89
NY-ESO1/2	0	0	0	0	0	0	1	1,9	0	0	3,6	0,52	13	9,2	0	0	3,6	0,68	64	12	0	0	5,6	0,92	19	27,5	0,2	0	5,4	1,23

TARGET	Expression MBC						Expression PPC						Expression HMCL					
	n	%	median	min	max	SDV	n	%	median	min	max	SDV	n	%	median	min	max	SDV
CD38	4	100	2,6	1,9	3	0,55	4	100	8,6	8,4	8,8	0,21	25	96,2	5,6	0,3	9,6	2,41
CS1	4	100	4,2	2,2	4,5	1,05	4	100	8,5	8,4	8,7	0,18	25	96,2	7,4	0,2	9,9	2,55
BCMA	4	100	2,1	1,3	2,5	0,56	4	100	9,2	8,8	9,5	0,33	26	100	7,7	5	9,8	1,35
GPRC5D	3	75	2	0,8	2,8	0,86	4	100	3,5	2,6	4,3	0,74	24	92,3	5,5	0	7,8	2,13
FCRH5	4	100	5	3,5	6,3	1,57	4	100	8	7,8	8,2	0,16	15	57,7	2,4	0	6,7	2,27
BAFFR	4	100	10,5	10,4	10,7	0,14	4	100	4,9	4,7	5,3	0,25	6	23,1	1,3	0,1	9,2	1,86
TACI	4	100	6	5,4	6,8	0,78	4	100	6,7	5,9	7,1	0,55	15	57,7	3,5	0	10	3,09
CD19	4	100	6,8	6	7,5	0,62	4	100	6,2	5,7	6,7	0,43	2	7,7	0,1	0	7,2	1,41
CD20	4	100	10	9,5	11,6	0,91	4	100	2,5	1,9	2,9	0,42	5	19,2	0,1	0	10,9	2,74
CD74	4	100	14,5	13,9	15	0,45	4	100	10,9	10,5	11,2	0,3	24	92,3	8,5	0,8	12,4	4,03
CD22	4	100	7,6	7	8,2	0,51	3	75	1,5	1	2,5	0,67	3	11,5	0,2	0	9,1	1,97
CD44	4	100	9,4	8,6	9,8	0,51	4	100	9,7	9,3	9,9	0,31	24	92,3	7	0,4	10,6	2,98
CD1B	0	0	0	0	0,1	0,07	0	0	0	0	0,1	0,02	0	0	0	0	0,1	0,02
CD25	4	100	3,8	2,7	4,4	0,84	4	100	2,6	1,2	3,7	1,07	1	3,8	0	0	2,7	0,53
CD30	0	0	0,1	0	0,1	0,06	0	0	0,9	0,1	1,4	0,54	14	53,8	2	0	6	1,48
CD37	4	100	9,8	8,9	10,2	0,6	4	100	5,9	5,3	6,4	0,53	16	61,5	3,3	0,1	7,6	2,33
CD70	4	100	6,2	4,6	6,3	0,8	4	100	4,6	4,4	5,3	0,42	23	88,5	5,9	0,3	9,5	2,66
CD79B	4	100	7,2	7	7,7	0,31	4	100	7,2	7,1	7,5	0,2	24	92,3	5,5	0	7,9	2,12
MUC1	0	0	0,2	0	0,5	0,21	2	50	0,9	0,5	1,3	0,37	8	30,8	0,5	0	2,5	0,81
NY-ESO1/2	0	0	0	0	0	0,02	0	0	0	0	0,2	0,09	17	65,4	3,2	0	6,2	2,41

<u>Supplementary Table S3</u>. <u>RNA-sequencing based assessment of differential presence of expression of 20 suggested immune-oncological actionable targets</u> between multiple myeloma cells (MMC) from MGUS, AMM, therapy requiring MM (MM), relapsed MM (MMR) [MGUS-AMM-MM-MMR], and AMM, MM, MMR [AMM-MM-MMR]. – denotes genes expressed in all patients for which no statistical comparison can be made. Chi-squared test for trend in proportion (Cochran-Armitage trend test). Exploratory *P*-values given.

presence of expression (trend)	CD38	CS1	BCMA	GPRC5D	FCRH5	BAFFR	TACI	CD19	CD20	CD74
AMM-MM-MMR	--	0.85	--	0.75	0.85	<0.001	<0.001	<0.001	<0.001	--
MGUS-AMM-MM-MMR	--	0.75	--	0.58	0.75	<0.001	0.0012	<0.001	<0.001	--

presence of expression (trend)	CD22	CD44	CD1B	CD25	CD30	CD37	CD70	CD79B	MUC1	NYESO
AMM-MM-MMR	0.0032	0.0646	0.0842	0.44	0.58	0.0326	0.11	--	0.0056	0.0013
MGUS-AMM-MM-MMR	<0.001	0.0521	0.26	0.0015	0.27	0.0432	0.66	--	0.0025	<0.001

Supplementary Table S4. RNA-sequencing based assessment of 20 suggested immune-oncological actionable targets (IOnc-advisor). Independent validation on a cohort of 767 symptomatic previously untreated myeloma patients (CoMMpass-cohort). Targets can be divided in those expressed in all normal and (almost all, >99%) malignant plasma cells (MMC; n=10, i.e., CD38, SLAMF7 (CS1), BCMA, GPRC5D, FCRH5, TACI, CD74, CD44, CD37 and CD79B), those constitutively expressed in all normal plasma cells with expression lost in a subfraction of malignant plasma cells (n=4, i.e., BAFF-R, CD19, CD20, CD22), and targets aberrantly expressed in malignant plasma cells (i.e., not expressed in BMPC; n=3, NY-ESO1/2, MUC1, CD30). CD70 is expressed in a subfraction of normal plasma cells in our cohort with decreasing expression frequency in multiple myeloma. Some suggested targets are not expressed (CD1B) or at a very low level in normal and malignant plasma cells (CD25). Given are median expression in normal and malignant plasma cells, %age of patients expressing the respective gene, and standard deviation (SDV) within the respective population, i.e., normal or malignant plasma cells. Note different expression height, e.g., detectable but low CD20 median expression.

TARGET	Expression MMC CoMMpass-cohort					
	n	%	median	min	max	variation
CD38	767	100	8.3	4.8	10.4	0.75
CS1	767	100	9.9	6.5	12.6	0.76
BCMA	767	100	7.8	4	13.4	1.03
GPRC5D	758	98.8	6.8	0.5	9.6	1.71
FCRH5	767	100	8.9	3.2	10.8	0.98
BAFFR	578	75.4	2.3	0	6.5	1.56
TACI	757	98.7	6.3	0.3	10.8	1.38
CD19	238	31	0.8	0	7.3	1.72
CD20	449	58.5	1.3	0	12.7	2.82
CD74	767	100	9.6	4.2	13.6	1.37
CD22	111	14.5	0.3	0	8.8	0.75
CD44v6	734	95.7	6.6	0.2	10.7	2.63
CD1b	0	0	0	0	0.5	0.03
CD25	13	1.7	0	0	4	0.36
CD30	10	1.3	0.1	0	5.3	0.55
CD37	765	99.7	5.3	0.6	7.9	1.1
CD70	106	13.8	0.2	0	7.4	0.82
CD79b	767	100	6.1	3.5	8.9	0.68
MUC1	421	54.9	1.2	0	7.3	1.4
NY-ESO1/2	161	21	0.1	0	7	1.33

Supplementary Table S5. Changes in target expression between previously untreated (MM) and relapsed multiple myeloma patients (MMR). Shown are data for 63 longitudinal patients. See also Figure 3 and Supplementary Table S6.

Target	MM %	MMR %	gain MMR %	loss MMR %	change %
CD38	100	100	0	0	0
CDS1	100	100	0	0	0
BCMA	100	100	0	0	0
GPRC5D	100	98	0	2	2
FCRH5	100	100	0	0	0
BAFFR	78	62	6	22	29
TACI	100	90	0	10	10
CD19	43	49	21	14	35
CD20	81	71	8	17	25
CD74	100	100	0	0	0
CD22	30	32	17	16	33
CD44	98	97	0	2	2
CD1B	0	0	0	0	0
CD25	2	0	0	2	2
CD30	6	6	5	5	10
CD37	100	97	0	2	2
CD70	16	27	21	10	30
CD79B	100	100	0	0	0
MUC1	10	21	16	5	21
NY-ESO1/2	6	27	24	3	27

Supplementary Table S6. Changes in median target expression between previously untreated (MM) and relapsed multiple myeloma patients (MMR). Shown are data for 63 longitudinal patients. See also Figure 3 and Supplementary Table S5. SDV standard deviation. *P*-adj adjusted P-value.

TARGET	Expression MM						Expression MMR						Expression Difference				Significance	
	n	%	median	min	max	SDV	n	%	median	min	max	SDV	median	max	min	SDV	*P* -value	*P*- adj.
**CD38**	**63**	100	8,5	6,4	10,9	0,86	**63**	100	8,5	6,5	10,3	0,78	0,5	1,8	0	0,44	ns	ns
**CS1**	**63**	100	9,7	7,6	11,5	0,79	**63**	100	9,7	6,6	11	0,87	0,3	1,9	0	0,49	ns	ns
**BCMA**	**63**	100	9	6	10,9	0,9	**63**	100	9	6,3	11,7	0,86	0,6	2,8	0	0,63	ns	ns
**GPRC5D**	**63**	100	8,1	1,8	10,4	1,69	**62**	98,4	7,2	0,7	10,6	1,89	0,9	3,9	0	0,84	<0.01	<0.05
**FCRH5**	**63**	100	8,3	4,4	10,4	1,13	**63**	100	7,7	3,4	9,8	1,14	0,7	6,7	0	0,97	<0.01	<0.05
**BAFFR**	**49**	77,8	4,5	0,2	8,5	2,25	**39**	61,9	3,3	0,1	8,6	2,29	1,3	5,8	0,1	1,39	<0.05	ns
**TACI**	**63**	100	6,8	1,8	9,2	1,5	**57**	90,5	6,7	0,2	8,8	2,2	0,7	8,4	0	1,59	<0.05	ns
**CD19**	**27**	42,9	1,2	0	8,1	2,47	**31**	49,2	1,5	0	7,8	1,92	1,1	5,4	0	1,38	ns	ns
**CD20**	**51**	81	3	0	11,2	3,28	**45**	71,4	2,4	0	11	3,44	1,6	7,7	0	1,71	ns	ns
**CD74**	**63**	100	10,6	7,6	15,6	1,55	**63**	100	10,5	5,7	13,3	1,56	0,8	4,3	0	1,02	<0.01	<0.05
**CD22**	**19**	30,2	0,5	0	9,5	1,35	**20**	31,7	0,5	0	8,7	1,33	0,4	2,7	0	0,75	ns	ns
**CD44**	**62**	98,4	6,8	0,6	10,4	2,66	**61**	96,8	6,3	0,4	10,3	2,64	1,4	6,5	0,1	1,55	ns	ns
**CD1B**	**0**	0	0	0	0,7	0,11	**0**	0	0	0	0,5	0,12	0,1	0,7	0	0,12	<0.05	ns
**CD25**	**1**	1,6	0,1	0	1,5	0,24	**0**	0	0,1	0	1	0,23	0,1	1,5	0	0,24	<0.01	<0.05
**CD30**	**4**	6,3	0,2	0	2,2	0,54	**4**	6,3	0,4	0	2,9	0,65	0,2	2,4	0	0,57	<0.05	ns
**CD37**	**63**	100	5,8	1,4	7,6	1,19	**61**	96,8	5,3	0,3	7,4	1,54	0,8	3,9	0,1	0,92	<0.001	<0.05
**CD70**	**10**	15,9	0,3	0	5	0,91	**19**	30,2	0,5	0	5,9	1,16	0,5	4,5	0	1,02	<0.05	ns
**CD79B**	**63**	100	6,8	5	9	0,88	**63**	100	6,7	5,4	8,3	0,68	0,6	1,7	0	0,42	ns	ns
**MUC1**	**6**	9,5	0,1	0	1,9	0,43	**13**	20,6	0,2	0	4,7	0,85	0,3	4	0	0,73	ns	ns
**NY-ESO1/2**	**5**	7,9	0	0	4,7	0,81	**17**	27	0,2	0	5,4	1,25	0,2	5,4	0	1,11	<0.001	<0.01

<u>Supplementary Table S7</u>. **Comparison and concordance of determination of proliferation, risk-stratification, and detection of target-expression by RNA-sequencing vs. GEP.** Depicted are % of patients defined as high / (medium) / low risk according to different risk scores and proliferation (RPI vs. GPI) (n=535) as assessed by RNA-sequencing vs. GEP.

Score	low risk in both	medium risk in both	high risk in both	overall concordance
G/RPI	26.6	43.3	9.6	79.4
UAMS70	72.1	NA	16.3	88.4
RS	18.4	49.1	8.2	75.7
SKY92	85.2	NA	7.3	92.5
LfM-HRS	33.0	22.8	15.9	71.7

SUPPLEMENTARY METHODS

HUMAN MYELOMA CELL LINES USED

The human myeloma cell lines (HMCL) L363, SK-MM-2, LP-1, OPM-2, U266, RPMI-8226, AMO-1, JJN3, KARPAS-620, KMS-12-BM, KMS-11, NCI-H929, MOLP-8, KMM-1, and EJM were purchased from the German Collection of Microorganisms and Cell Cultures (Braunschweig, Germany), American Type Cell Culture (Wesel, Germany), or the National Institutes of Biomedical Innovation, Health and Nutrition (Osaka, Japan); the HG-lines HG1, HG13, and HG19 were generated at the Myeloma Research Laboratory Heidelberg (Germany), the XG1, XG2, XG3, XG4, XG6, XG7, XG11, and XG13 at the CHU Montpellier, France. Cell line identity was assessed for proprietary cell lines by DNA-fingerprinting, mycoplasma-contamination excluded by PCR-based assays, and EBV-infection status by clinical routine PCR-based diagnostics.

STATISTICAL ANALYSIS OF GEP AND RNA-SEQUENCING DATA

GEP analyses were performed on GC-RMA[1] preprocessed data as previously published[2,3]. Presence or absence of gene expression was determined using the "Presence-Absence calls with Negative Probesets" (PANP) algorithm[4]. The UAMS70 -score[5], RS-score[6] , SKY92-score[7], and gene expression-based proliferation index (GPI)[8] have been calculated as published (see also below). For calculation of the UAMS70-score data were normalized using the mas5-algorithm (Affymetrix).

RNA-sequencing analysis. RNA fastq-files were aligned with STAR with default options[9]. Files were aligned to GRCh38 genome build and reads were counted per gene. STAR uses HTSeq[10] internally for counting reads. Technical replicated were summed and reads per gene were normalized with edgeR[11]. "Present" expression by RNA-sequencing is defined as presence of at least one read count per million (CPM) per 1000 bp. Gene length is defined as median transcript length. For BRAF V600E/K mutation reads were counted per base and filtered using bam-readcount (minimum 2 reads covering the mutation, mapping quality >= 255, base quality >= 30, at least one read in each

strand direction, an average base position in the intermediate 85% of the nucleotides and a VAF >= 10%).

For general mutation analysis, single nucleotide variants (SNVs) were determined using the h5vc R package. For this, a HDF5 tally file was prepared per gene for all patients, using the functions prepareTallyFile and batchTallies and SNVs were called with the callVariantsSingle function with default parameters. Variants were annotated with vep, filtered for present (variant allele frequency ≥0.1), coding and non-synonymous SNVs and merged per patient.

NY-ESO-1/2 expression analysis. Due to high sequence homology of CTAG2, CTAG1A, CTAG1B, coding for NY-ESO1 and NY-ESO2, the maximal gene expression values of these three genes was used as previously described[12].

PROLIFERATION AND HIGH-RISK SCORES – IMPLEMENTATION AND VALIDATION

Validation strategy. The cohort was divided in three groups. First, a dataset with four MBC, four PPC, nine BMPC, 26 MGUS, 26 HMCL, 194 symptomatic and 19 asymptomatic myeloma patient samples was used for compiling a training group (TG), termed TG 1. TG 2 comprises only the 194 symptomatic myeloma patient samples from TG 1. TG 1 was used for GPI (see below) and t(4;14) related analyses, TG 2 for all other scores created on symptomatic MM patient only. The validation group (VG) with 108 symptomatic myeloma samples was used for internal comparison and validation of survival performances of the scores. A test group (TeG) with 233 symptomatic myeloma samples was used to independently validate the scores. Additionally, all scores have been independently validated on the external CoMMpass cohort (Supplementary Figure S2, S4). Validation of scores was performed by examining the proportion of the classes, matching original GEP-scores, and comparing performance of survival analysis.

Normalization strategy. DNA-microarray normalization was performed for TG with GC-RMA[1], saving the normalization parameters. The "documentation by value" (docval) strategy[13] [https://storage.googleapis.com/google-code-archivedownloads/v2/code.google.com/gep-r/docval_1.1.2_gcrma.tar.gz] was used to normalize VG and TeG with the parameters of the TG. This approach enables comparability to former normalized samples, which is the basis for use in clinical trial and routine setting. Hence this strategy was adopted for RNA-sequencing approach.

Before RNA-sequencing normalization, genes with no counts in all samples were excluded from the TG. Normalization was performed with edgeR[11] as previously published[2,14]. For the samples of the VG and TeG, each new sample is added to the TG and jointly normalized. Normalization was performed for each sample of VG and TeG twice, first with TG 1, secondly with TG 2.

As the external CoMMpass cohort was aligned against the (older) human genome GRCh37, the cohort was normalized separately with edgeR. The expressions were standardized using a modified Z-score normalization, including mean and standard deviation of the CoMMpass and a subset of the LfM-cohort. The subset includes samples of patient with a similar age distribution as the CoMMpass cohort.

Implementation of RNA-sequencing based risk scores. Probesets or gene IDs of GEP-based scores were translated to Ensembl gene identifiers (ENSG) in R using the annotation Database hgu133plus2.db[15]. Genes with missing translation were excluded. Expression values of probesets matching to more than one ENSG are added. An ENSG is only used once, even if it matches to several probesets, and associated values are averaged. Genes with a correlation $r \leq 0.15$ between RNA-sequencing and DNA-microarray expression were excluded. For genes with a correlation $r \leq 0.6$, the translation was controlled using the GeneCards database[16]. If the translation is inconsistent to the annotation Database, the gene was excluded. Further, genes with correlation $r \leq 0.4$ and percentage

of samples with absent expression in RNA-sequencing and present expression in DNA-microarrays > 30% were excluded.

UAMS70-, SKY92-, and RS-score were calculated as described for microarray, transferred to RNA-sequencing data. All score calculations were kept as similar as possible to the original GEP-based scores, using the microarray based values as weighting scores, expression averages, and factors (UAMS70 -score[5], RS-score[6] , and SKY92-score[7]). Cutoffs for classification were recalculated on RNA-sequencing expression data for each score, following the published description for microarray-based scores.

RNA-sequencing proliferation index. For assessing proliferation, 50 proliferation associated genes[8] were analyzed. Genes with expression less than one normalized count per 1000 bp were excluded. A continuous score was calculated analogous to GPI on microarray by summing the expression of the remaining genes. Two cutoffs were determined by correlating the continuous GPI[8] on DNA-microarrays and RNA-sequencing, fitting a linear regression line, and transferring the cutoffs.

LfM HRS. The method published by Rème et al.[6] was used to generate a novel RNA-sequencing based risk stratification. This method performs three steps after normalization. First, 53 prognostic genes were selected using a running log-rank test. For each gene a prognostic factor was determined, which is 1 in case high expression is associated with poor prognosis and -1 in the opposite case. Second, 53 genes were used to calculate the score by multiplying each expression value with its prognostic factor and summing up the values. Third, the multi-cutoff running log -rank algorithm was used for risk group optimization, resulting in two cutoffs. The samples were stratified in three groups.

REFERENCES

1. Wu Z, Irizarry RA, Gentleman R, Martinez-Murillo F, Spencer F. A Model-Based Background Adjustment for Oligonucleotide Expression Arrays. *J Am Stat Assoc*. 2004;99:909-917.
2. Seckinger A, Delgado JA, Moser S, et al. Target Expression, Generation, Preclinical Activity, and Pharmacokinetics of the BCMA-T Cell Bispecific Antibody EM801 for Multiple Myeloma Treatment. *Cancer Cell*. 2017;31(3):396-410.
3. Hose D, Beck S, Salwender H, et al. Prospective target assessment and multimodal prediction of survival for personalized and risk-adapted treatment strategies in multiple myeloma in the GMMG-MM5 multicenter trial. *J Hematol Oncol*. 2019;12(1):65.
4. Warren P, Taylor D, Martini PGV, Jackson J, Bienkowska J. PANP - a New Method of Gene Detection on Oligonucleotide Expression Arrays. Proceedings of the 7th IEEE International Conference on Bioinformatics and Bioengineering; 2007:108-115.
5. Shaughnessy JD, Jr., Zhan F, Burington BE, et al. A validated gene expression model of high-risk multiple myeloma is defined by deregulated expression of genes mapping to chromosome 1. *Blood*. 2007;109(6):2276-2284.
6. Reme T, Hose D, Theillet C, Klein B. Modeling risk stratification in human cancer. *Bioinformatics*. 2013;29(9):1149-1157.
7. Kuiper R, Broyl A, de Knegt Y, et al. A gene expression signature for high-risk multiple myeloma. *Leukemia*. 2012;26(11):2406-2413.
8. Hose D, Reme T, Hielscher T, et al. Proliferation is a central independent prognostic factor and target for personalized and risk-adapted treatment in multiple myeloma. *Haematologica*. 2011;96(1):87-95.
9. Dobin A, Davis CA, Schlesinger F, et al. STAR: ultrafast universal RNA-seq aligner. *Bioinformatics*. 2013;29(1):15-21.
10. Anders S, Pyl PT, Huber W. HTSeq--a Python framework to work with high-throughput sequencing data. *Bioinformatics*. 2015;31(2):166-169.
11. Robinson MD, McCarthy DJ, Smyth GK. edgeR: a Bioconductor package for differential expression analysis of digital gene expression data. *Bioinformatics*. 2010;26(1):139-140.
12. Schmitt M, Huckelhoven AG, Hundemer M, et al. Frequency of expression and generation of T-cell responses against antigens on multiple myeloma cells in patients included in the GMMG-MM5 trial. *Oncotarget*. 2017;8(49):84847-84862.
13. Kostka D, Spang R. Microarray based diagnosis profits from better documentation of gene expression signatures. *PLoS Comput Biol*. 2008;4(2):e22.
14. Seckinger A, Hillengass J, Emde M, et al. CD38 as Immunotherapeutic Target in Light Chain Amyloidosis and Multiple Myeloma-Association With Molecular Entities, Risk, Survival, and Mechanisms of Upfront Resistance. *Front Immunol*. 2018;9:1676.
15. Carlson M. hgu133plus2.db: Affymetrix Human Genome U133 Plus 2.0 Array annotation data (chip hgu133plus2). R package version 3.2.3. *https://bioconductororg/packages/34/data/annotation/html/hgu133plus2dbhtml*. 2016.
16. Stelzer G, Rosen N, Plaschkes I, et al. The GeneCards Suite: From Gene Data Mining to Disease Genome Sequence Analyses. *Curr Protoc Bioinformatics*. 2016;54:1.30.31-31.30.33.
